# Jiegeng decoction ameliorates COPD-like pulmonary injury in association with changes in PPARγ expression, arachidonic acid metabolism, and gut microbial composition

**DOI:** 10.3389/fphar.2026.1933011

**Published:** 2026-10-28

**Authors:** Xinhai Cui, Xiaowen Sun, Fangjie Li, Kejian Li, Xianjun Fu, Kuo Xu

**Affiliations:** 1 Key Laboratory of Marine Traditional Chinese Medicine in Shandong Universities, Research Institute for Marine Traditional Chinese Medicine (Qingdao Academy of Chinese Medical Sciences), The SATCM’s Key Unit of Discovering and Developing New Marine TCM Drugs, Shandong University of Traditional Chinese Medicine, Qingdao, China; 2 College of Traditional Chinese Medicine, Shandong University of Traditional Chinese Medicine, Jinan, China

**Keywords:** arachidonic acid metabolite, chronic obstructive pulmonary disease, gut microbiota, Jiegeng decoction, PPARγ

## Abstract

**Background:**

The Jiegeng Decoction (JGD) is a classic traditional Chinese medicinal formulation that consists of *Platycodon grandiflorus* (Jacq.) A. DC. and *Glycyrrhiza uralensis* Fisch. This formulation was first recorded in the “Treatise on Cold Damage and Miscellaneous Diseases” during the Eastern Han Dynasty. Clinically, JGD has been employed as a fundamental prescription in the clinical management of various pulmonary disorders. Unlike previous studies, which focused primarily on the addition and subtraction prescriptions derived from JGD, the present study first sought to investigate the associated mechanisms and identify the principal bioactive metabolites of this foundational prescription for the treatment of chronic obstructive pulmonary disease (COPD)-like pulmonary injury, with particular attention to its underexplored effects on the intestine.

**Methods:**

A cigarette smoke/lipopolysaccharide (LPS)-induced rat model of COPD-like pulmonary injury was used to assess the effects of JGD on pulmonary and intestinal injury. This assessment utilized histological staining, immunofluorescence, and enzyme-linked immunosorbent assays. To explore and validate the underlying mechanisms and bioactive metabolites, lung transcriptomics, untargeted metabolomics, 16S rRNA sequencing, UPLC‒Q‒Orbitrap‒MS/MS, and LPS-stimulated RAW264.7 macrophages were utilized.

**Results:**

An animal study revealed that JGD mitigated pulmonary histopathological damage, mucus hypersecretion, and the release of inflammatory cytokines in rats exposed to cigarette smoke/LPS-. Comprehensive transcriptomic and metabolomic analyses suggested that JGD may ameliorate inflammatory responses by modulating arachidonic acid metabolism, potentially through the upregulation of PPARγ expressions. Additionally, JGD alleviated intestinal injury and inflammation, restored the expressions of the barrier-associated proteins MUC2 and ZO-1, and was associated with alterations in the composition of the gut microbiota. Notably, specific microbial taxa were significantly correlated with pathological indicators and arachidonic acid metabolites. A total of 156 JGD-derived metabolites were detected and annotated in JGD, 77 of which were absorbed into the bloodstream. Among these compounds, liquiritigenin (LG) has emerged as a potential bioactive metabolite that can interact with PPARγ. *In vitro* studies demonstrated that both JGD and LG mitigated LPS-induced inflammation in RAW264.7 cells by restoring PPARγ expressions. Importantly, pharmacological inhibition of PPARγ using GW9662 significantly reduced these protective effects, supporting the involvement of PPARγ signaling in LG-mediated inflammatory regulation.

**Conclusion:**

JGD may mitigate COPD-like pulmonary injury through a multitarget approach associated with the upregulation of PPARγ expressions, the modulation of arachidonic acid metabolism, the restoration of intestinal barrier-associated protein expression, and changes in the gut microbial composition. The LG within JGD may serve as a bioactive metabolite that contributes to these therapeutic effects.

## 1 Introduction

Chronic obstructive pulmonary disease (COPD) remains a leading cause of global mortality, significantly impairing patient quality of life and life expectancy (Shahbazi et al., 2025). Clinically characterized by persistent airflow limitation, the disease is driven by persistent pulmonary inflammation and structural tissue damage (Goyal et al., 2025). Furthermore, this inflammatory cascade triggers mitochondrial dysfunction, dysregulated autophagy, cellular senescence, and abnormal fibrin deposition, collectively accelerating disease progression (Górski et al., 2024; Wang et al., 2024a). Current conventional therapies, including inhaled long-acting β2-agonists, muscarinic antagonists, and corticosteroids, primarily aim to alleviate symptoms and prevent exacerbations (Jacques et al., 2024; Wu et al., 2024). However, these pharmacological interventions offer limited efficacy in halting disease progression and are associated with various adverse effects, including cardiovascular and metabolic disturbances. Therefore, identifying safer and more effective therapeutic strategies is needed.

Traditional Chinese medicine (TCM) has been evaluated as a complementary therapeutic approach for the management of chronic disorders (Li et al., 2024). Jiegeng Decoction (JGD), first documented in China’s classic medical text “Treatise on Cold Damage and Miscellaneous Diseases,” is composed of two botanical drugs: *P. grandiflorus* (Jacq.) A. DC. and *Glycyrrhiza uralensis* Fisch. (Liu et al., 2019). As a prominent TCM formula, owing to its ability to promote expectoration, detoxification, and lung nourishment and alleviate throat discomfort, JGD is extensively used in clinical practice for the treatment of respiratory diseases (Wu et al., 2023). Previous studies have shown that JGD has protective effects on pulmonary disorders; notably, it can alleviate bleomycin-induced pulmonary fibrosis, and integrated lipidomic and transcriptomic analyses have revealed that the PI3K/Akt pathway and apoptosis processes are involved in its mechanism of action (Xu et al., 2022). In addition, an earlier study using modified JGD in COPD model rats revealed improvements in both pulmonary and intestinal pathological injuries, suggesting that modified JGD may affect lung‒gut interactions in patients with COPD (Wang, 2011). Moreover, recent evidence from *Platycodon grandiflorus*-derived polysaccharides and saponins has further indicated that these interventions can ameliorate COPD by repairing the intestinal barrier, remodeling the gut microbiota, and promoting microbiota-mediated biotransformation through the gut–lung axis (Xu et al., 2025). Nevertheless, studies directly focusing on JGD in COPD are still limited, and the bioactive metabolites, the integrated mechanisms linking molecular targets and host metabolism, and the gut microbiota involved have not yet been systematically elucidated. This gap, particularly the poorly characterized intestinal effects of JGD, provides a rationale for further investigations of its bioactive metabolites and mechanisms in COPD.

To address these gaps, the present study is the first to combine an *in vivo* pharmacological evaluation, transcriptomics, metabolomics, and 16S rRNA sequencing to systematically investigate the therapeutic mechanisms of JGD against COPD-like pulmonary injury. On this basis, UPLC‒Q-Orbitrap‒MS/MS-based chemical characterization and *in vitro* experiments in LPS-stimulated RAW264.7 macrophages were performed to identify JGD-derived metabolites, validate key mechanistic findings, and pinpoint candidate active constituents. This study provides multitargeted mechanistic evidence supporting the anti-COPD-like pulmonary injury effects of JGD and facilitates its further development as a phytotherapeutic strategy.

## 2 Materials and methods

### 2.1 Preparation of intervention reagents

*P*. *grandiflorus* (Campanulaceae; Platycodonis Radix) and *G*. *uralensis* (Fabaceae; Glycyrrhizae Radix et Rhizoma) were purchased from the Banger Traditional Chinese Medicine Co., Ltd. (Jining, China; batch No. 202201271) and from Qunkang Pharmaceutical Co., Ltd. (Anhui, China; batch No. 20180395). The botanical identities of the botanical drugs were authenticated by Feng Li of Shandong University of Traditional Chinese Medicine. LPS was obtained from Solebao Technology Co., Ltd. (China). Liquiritigenin (LG) was obtained from Aladdin (Shanghai, China). GW9662 was obtained from MedChemExpress (Shanghai, China). Dexamethasone (DXM) was obtained from Xianju Pharmaceutical Co., Ltd. (Zhejiang, China).

As mentioned previously (Mao et al., 2017), 240 g of *P*. *grandiflorus* and 480 g of *G*. *uralensis* were decocted in 10 L of water for 30 min before a second decoction with 8 times the volume of water. After being filtered and concentrated, JGD was obtained at an ultimate concentration of 2 g/mL. The dry extract yield was 32.17%. The chemical quality of JGD was evaluated using chromatographic fingerprinting and quantitative determination of representative marker metabolites. The JGD-derived metabolites were characterized by UPLC‒Q-Orbitrap‒MS/MS, with detailed information provided in the Supplementary Methods.

### 2.2 Animal experiments

Six-week-old male Sprague–Dawley (SD) rats were obtained from Beijing Vital River Laboratory Animal Technology Co., Ltd. [SCXK (Jing) 2021–0011]. After acclimatization, each animal was assigned a unique identification number, and the rats were randomly allocated to five experimental groups using a computer-generated random-number sequence (n = 6 per group): control, model, low-dose JGD (JGD-L), high-dose JGD (JGD-H), and dexamethasone (DXM). To establish the COPD-like pulmonary injury model, the rats smoked within a controlled compartment twice daily (20 min/session), with a minimum 3-h interval between sessions. Additionally, 1 mg/kg LPS was administered intratracheally at 1-week intervals. The control and model groups received 0.9% NaCl by gavage, whereas the JGD-L and JGD-H groups received 0.81 and 2.43 g/kg/day JGD, respectively, and the DXM group received 0.135 mg/kg/day dexamethasone. The JGD doses were selected with reference to a previous pharmacological study of JGD in rats (Huang et al., 2024). All animals received daily treatments and were euthanized after 56 days by inhalation of an overdose of isoflurane (5% in oxygen, administered once). Blood, lung, and colon samples were subsequently collected for further assays. The number of independent biological replicates used for each assay, together with the detailed allocation procedure and exclusion criteria, is provided in the Supplementary Methods.

### 2.3 Pathological morphology analysis

Left lung and colon samples were fixed in 4% paraformaldehyde for 24 h, embedded in paraffin, and cut into 5-μm serial sections. Hematoxylin and eosin (H&E) staining and alcian blue-periodic acid-Schiff (AB-PAS) staining were then performed. Images were acquired and analyzed using a ZEISS microscope (Germany). Histopathological scoring and quantitative image analyses were performed by investigators who were blinded to group allocations using coded tissue sections and images.

### 2.4 Enzyme-linked immunosorbent assay (ELISA)

The levels of inflammatory cytokines and key enzymes involved in arachidonic acid metabolism were measured by ELISA in accordance with the protocol. Detailed information is described in the Supplementary Methods.

### 2.5 Serum-detected JGD-derived prototype metabolites

Six male SD rats were randomly divided into two groups, the blank group and the JGD group, with three rats in each group. The rats in the JGD group were orally administered JGD at 2.43 g/kg once daily for 1 week, while the rats in the blank group received an equivalent volume of 0.9% NaCl. At 1 h after the final gavage, blood samples were collected and centrifuged at 3,500 rpm for 10 min at 4 °C to obtain serum samples. The serum samples were mixed with methanol for protein precipitation, vortexed, ultrasonically extracted, and centrifuged at 12,000 rpm for 10 min at 4 °C. The supernatants were evaporated under nitrogen, reconstituted in 50% methanol, and analyzed by UPLC‒Q-Orbitrap‒MS/MS. The detailed chromatographic and mass spectrometric conditions are provided in the Supplementary Methods.

### 2.6 Transcriptomic analyses of lung tissue

Nine lung tissue samples were selected for high-throughput RNA sequencing. Detailed information is provided in the Supplementary Methods. From the gene expression results, the genes whose expressions differed among groups were filtered (fold change ≥1.2 and *P* < 0.05). Because no multiple-testing correction was applied at the gene-screening stage, these genes were treated as exploratory candidates rather than statistically significant differentially expressed genes. The resulting candidate gene sets were used for KEGG and GO enrichment analyses and pathway prioritization.

### 2.7 Untargeted serum metabolomics analysis

Serum metabolomic profiling was performed on an Agilent 1290 Infinity LC system (Agilent, USA) coupled to an AB SCIEX Triple TOF 6600 mass spectrometer (AB Sciex, USA). The detailed parameters are given in the Supplementary Methods. The raw data were processed with ProteoWizard (v3.0.6428) for metabolite identification. Candidate differentially abundant metabolites were selected using VIP >1.0 and one-way ANOVA (P < 0.05), and pathway assignments were performed with Omicsmart (http://www.omicsmart.com).

### 2.8 16S rRNA gene sequencing and analysis

When the experiment ended, rat feces were collected under aseptic conditions and then stored at −80 °C. The DNA of the intestinal content samples was extracted using a kit. Subsequently, polymerase chain reaction (PCR) amplification was executed on the total genomic DNA through primers specific to the V3-V4 areas of 16S: F: CCTACGGGNGGCWGCAG and R: GGACTACHVGGGTATCTAAT. Paired-end sequencing of the community DNA segments was implemented on the Illumina platform. First, the primer fragment of the sequence and the unmatched primer sequence were removed by QIIME cutadapt trim-pair. Afterward, operational taxonomic units (OTUs) with 97% sequence similarity were identified through Usearch (v 11.0.667). Representative sequences were annotated using SILVA (version 138.2) via the RDP classifier. Linear discriminant analysis effect size (LEfSe) analysis was employed to recognize the differential genera of the microbiota. All further data analyses were implemented through Omicsmart (http://www.omicsmart.com).

### 2.9 Target prediction and integrated omics analysis

Potential targets of the 77 serum-detected JGD-derived prototype metabolites were predicted via a multiplatform strategy. The canonical SMILES of each metabolite were retrieved from PubChem (https://pubchem.ncbi.nlm.nih.gov/) and used as query identifiers across the following three databases: (i) DrugBank (version 6.0, https://go.drugbank.com/); (ii) Therapeutic Target Database (TTD, 2024 version, https://idrblab.org/ttd/); and (iii) SwissTargetPrediction (2019 version, http://www.swisstargetprediction.ch/), where canonical SMILES were individually submitted with the species set to “*Rattus norvegicus*”, and predicted targets with probability scores >0.5 were retained. All the predicted targets from the three databases were pooled, and the gene names were standardized to official gene symbols using the UniProt Knowledgebase (https://www.uniprot.org/). Exploratory integration of the transcriptomic and metabolomic datasets was performed to prioritize shared candidate pathways, followed by Spearman correlation analysis to assess the associations among targets, metabolites, and pulmonary pathological and inflammatory indicators. The results were visualized using Omicsmart (http://www.omicsmart.com).

### 2.10 Real-time quantitative polymerase chain reaction (RT‒qPCR) analysis

Total RNA was isolated from lung tissues with a SPARKeasy RNA extraction kit (AC0202, Sparkjade). cDNA was synthesized using the SPARKscript II RT Plus kit (AG0304; Sparkjade), and RT‒qPCR was performed with SYBR Green Master Mix (AH0104; Sparkjade) on a Gentier 96E system (Tianlong, China). The primer sequences are listed in Supplementary Table S3. Relative mRNA expressions were calculated by the 2^−ΔΔCT^ method using β-actin as the reference gene.

### 2.11 Cell culture and treatment

RAW264.7 cells were obtained from the National Collection of Authenticated Cell Cultures and maintained in DMEM supplemented with 10% fetal bovine serum, penicillin, and streptomycin at 37 °C in 5% CO_2_. Cell viability following exposure to JGD or LG was evaluated using MTT assays. Cells were pretreated with JGD (50, 100, or 200 μg/mL) or LG (3.125, 6.25, or 12.5 µM) for 2 h and then challenged with LPS (1 μg/mL) for 24 h. For the inhibition experiments, GW9662 (10 µM) was added 3 h before LG treatment. JGD concentrations of 50, 100, and 200 μg/mL were selected on the basis of the cell viability assessment and concentration-dependent cellular responses, while the higher concentration of 400 μg/mL, which significantly reduced the measured viability signal, was excluded from subsequent experiments. Similarly, LG concentrations of 3.125, 6.25, and 12.5 μM were selected on the basis of cell viability and concentration-response assessments. The selected concentrations were used to investigate the effects of JGD and LG on LPS-induced inflammatory responses and PPARγ-related signaling in RAW264.7 macrophages.

### 2.12 Immunofluorescence staining

For tissue immunofluorescence determinations, the sections were dewaxed, rehydrated, subjected to antigen retrieval, blocked with 5% serum, and incubated overnight at 4 °C with primary antibodies against ZO-1 (21773-1-AP; Proteintech; 1:100) and MUC2 (27675-1-AP; Proteintech; 1:100). After being washed, the sections were incubated with secondary antibodies and counterstained with DAPI. RAW264.7 cells were cultured with appropriate primary antibodies [PPARγ antibody (16643-1-AP, Proteintech, 1:100)] and secondary antibodies after fixation, permeabilization, and blocking. After they were rinsed with PBS, the nuclei were coated with DAPI for 10 min. Images were captured with a fluorescence microscope (ZEISS, Germany).

### 2.13 Western blot analysis

Protein lysates from cells and tissues were prepared with cold RIPA buffer. Equal amounts of protein were separated by SDS‒PAGE and transferred onto polyvinylidene fluoride (PVDF) membranes (Millipore, USA). The membranes were blocked in TBST containing 5% skim milk for 1 h and incubated overnight at 4 °C with PPARγ antibody (16643-1-AP; Proteintech, 1:1000) or β-actin antibody (10494-1-AP; Proteintech, 1:20,000). Secondary antibody incubation was performed for 1.5 h at room temperature.

### 2.14 Molecular docking

First, the bidimensional structures of the JGD-derived prototype metabolites originated in PubChem. The receptor protein PPARγ (PDB: 3E00) was selected from the PDB (https://www.rcsb.org/). To ensure docking accuracy, active pockets on the proteins were identified and annotated as key docking sites. The CDOCKER algorithm in the receptor–ligand interaction unit of Discovery Studio 2019 R2 was employed for precise docking and analysis of metabolites with target proteins. The binding activity between receptors and ligands was evaluated on the basis of binding energy values.

### 2.15 Statistical analysis

The data are presented as the means ± SDs and were analyzed using GraphPad Prism 9.0 (USA). The normality and homogeneity of variance were tested before group comparisons. For comparisons among three or more groups, normally distributed data with equal variance were analyzed by one-way ANOVA followed by Tukey’s test; otherwise, the Kruskal‒Wallis test followed by Dunn’s test was applied. P < 0.05 was considered to indicate statistical significance.

## 3 Results

### 3.1 JGD attenuates cigarette smoke/LPS-induced COPD-like pulmonary injury in rats

To investigate the effects of JGD on alleviating pulmonary injury, rats were subjected to smoke and LPS to induce COPD-like pulmonary injury. The rats were then treated with 0.9% NaCl, JGD-L, JGD-H, or DXM (Figure 1A). As indicated in Figure 1B, the body weights dramatically decreased in smoke/LPS-exposed rats compared with those in healthy rats, whereas they increased significantly following treatment with JGD. Histopathological examination of lung tissue by H&E staining revealed that the model group displayed marked pathological damage, including inflammatory infiltration, alveolar septal thickening, and enlarged or destroyed alveolar spaces (Figures 1C,D). Both JGD-L and JGD-H alleviated these pathological changes, with the high-dose group exhibiting a more obvious protective effect, which was comparable to that of DXM (Figures 1C,D). To evaluate airway mucus secretion, AB‒PAS staining was performed. As shown in Figures 1E,F, JGD treatment reduced mucus accumulation to different extents in smoke/LPS-exposed rats, and the inhibitory effect was stronger in the high-dose group. As shown in Figure 1G, the bronchoalveolar lavage fluid (BALF) levels of tumor necrosis factor-α (TNF-α), interleukin-6 (IL-6), and interleukin-1β (IL-1β) markedly increased after modeling but decreased with JGD treatment, with a stronger response in the high-dose group. Overall, these data indicate that JGD effectively attenuated pulmonary inflammation, mucus hypersecretion, and histopathological injury in smoke/LPS-exposed rats.

**FIGURE 1:**
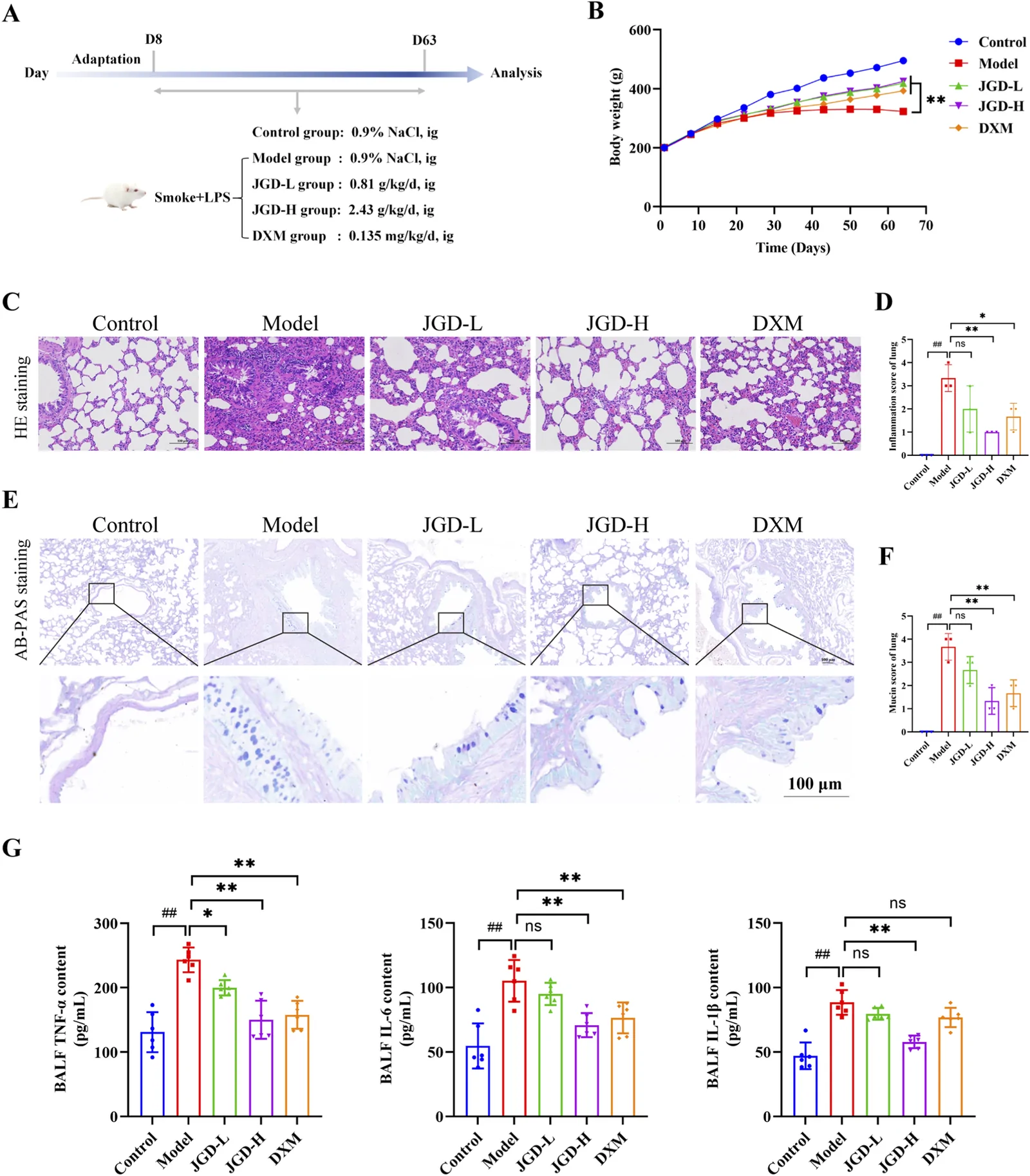
JGD alleviates cigarette smoke/LPS-induced COPD-like pulmonary injury in rats. **(A)** Sequence and duration of the animal assay. **(B)** Body weights of the indicated groups (n = 6 animals per group). **(C)** Representative images of H&E-stained lung sections (n = 3 animals per group). Scale bar: 100 μm. **(D)** Statistical analysis of the inflammation score determined by HE staining (n = 3 animals per group). **(E)** Representative images of AB-PAS-stained lung sections (n = 3 animals per group). Scale bar: 100 μm. **(F)** Statistical analysis of the AB-PAS staining score (n = 3 animals per group). **(G)** Inflammatory cytokine contents in the bronchoalveolar lavage fluid (BALF) (n = 6 animals per group). Body weight data **(B)** were analyzed by two-way repeated-measures ANOVA (group × time), followed by Bonferroni’s *post hoc* test. The data are presented as the means ± SDs and were analyzed by one-way ANOVA followed by Tukey’s multiple-comparisons test **(D,F,G)**. Relative to the control group, ^##^ *P* < 0.01; relative to the model group, ^*^ *P* < 0.05, ^**^ *P* < 0.01, ns: *P* > 0.05.

### 3.2 Transcriptomic analysis identifies PPAR signaling as a key pathway associated with JGD treatment in the lungs of smoke/LPS-exposed rats

RNA sequencing was conducted using lung tissues from the control, model, and JGD-H groups. Compared with the control group, the model group presented 1511 upregulated and 1498 downregulated DEGs, whereas compared with the model group, the JGD-H treatment group presented 626 upregulated genes and 783 downregulated genes (Figures 2A,B). Venn analysis revealed 928 overlapping DEGs between the control-vs.-model and model-vs.-JGD-H comparisons (Figure 2C). KEGG enrichment analysis indicated that these genes were associated mainly with metabolic pathways, with PPAR signaling among the prominently enriched pathways (Figure 2D). Further mapping of the DEGs onto the KEGG PPAR signaling pathway indicated that the altered genes were distributed mainly in the PPARγ-associated branch, involving multiple genes related to lipid uptake and fatty acid oxidation, such as *Fat/Cd36*, *Fabp*, *Fabp3*, *Lpl*, *Acs*, *Olr1*, *Cpt-1*, *Cpt-2*, *Lcad*, *aP2*, *Gyk*, and *Aqp7* (Figure 2E). To validate the sequencing results, representative genes in the PPAR pathway were further examined by RT‒qPCR. As shown in Figure 2F, the mRNA expression levels of *Pparg*, *Fat/Cd36*, *Cpt-1*, *Cpt-2*, *Lcad*, and *aP2* markedly decreased in the model group compared with those in the control group. JGD-H treatment significantly restored the expressions of these genes. These findings indicate that JGD-H markedly modulates the pulmonary transcriptomic profile and suggest that the restoration of PPARγ-centered lipid metabolic signaling may contribute to its protective effects.

**FIGURE 2:**
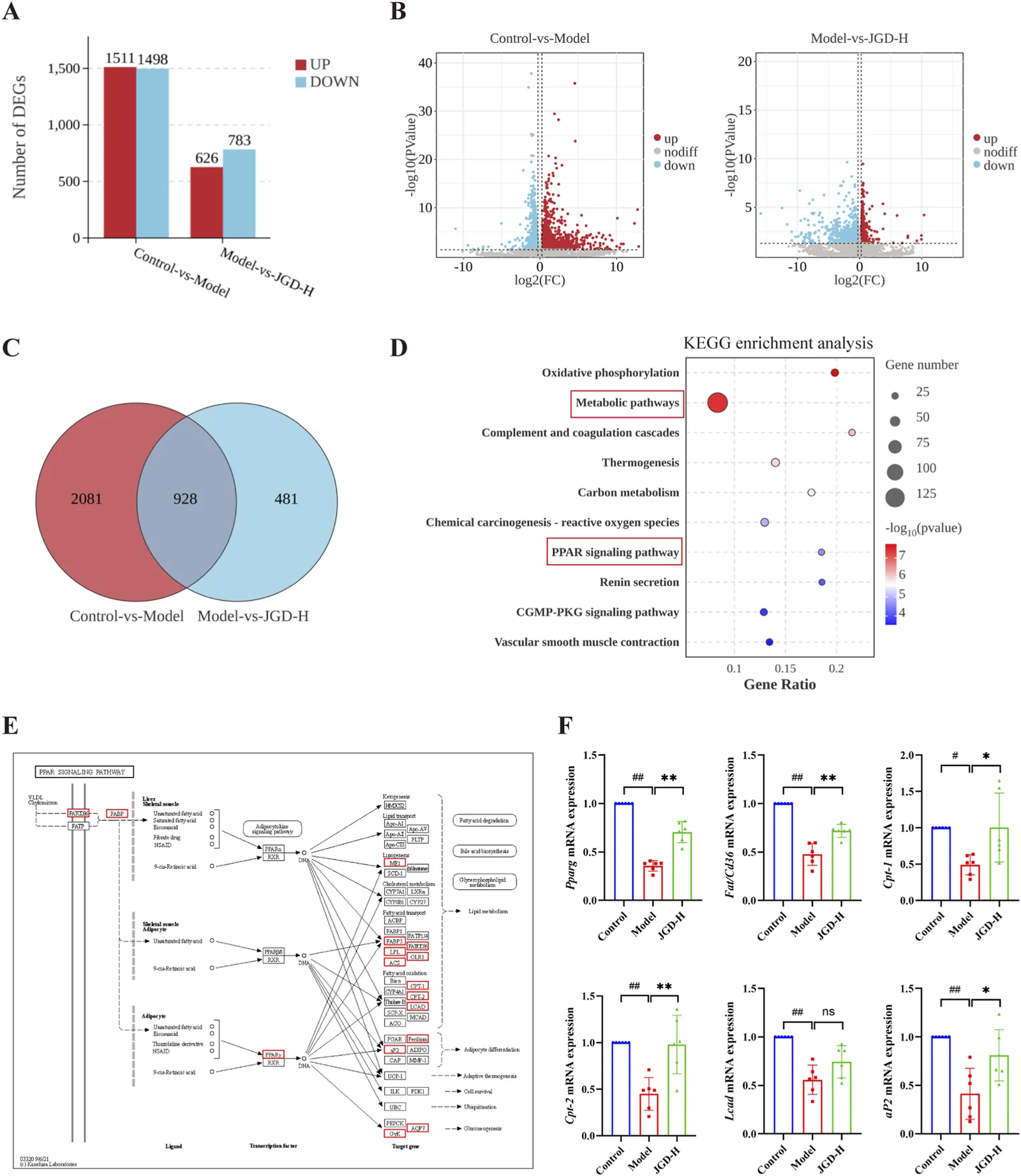
Transcriptomic analysis of lung tissues from smoke/LPS-exposed rats treated with JGD. **(A)** Bar plot of differentially expressed genes (DEGs) in the control-vs.-model and model-vs.-JGD-H comparisons. **(B)** Volcano plot of DEGs in the control-vs.-model and model-vs.-JGD-H comparisons. **(C)** Venn diagram of DEGs in the control-vs.-model and model-vs.-JGD-H comparisons. **(D)** Bubble plot of the top 10 KEGG signaling pathways. **(E)** Mapping of the overlapping DEGs onto the PPAR signaling pathway. Red boxes indicate DEGs altered after smoke/LPS exposure and reversed by JGD-H treatment. **(F)** Relative mRNA expression of DEGs in the control, model, and JGD-H groups, including *Pparg*, *Fat/Cd36*, *Cpt-1*, *Cpt-2*, *Lcad*, and *aP2* (n = 3 animals per group). The data are presented as the means ± SDs and were analyzed by one-way ANOVA followed by Tukey’s multiple-comparisons test **(F)**. Relative to the control group, ^#^ *P* < 0.05, ^##^ *P* < 0.01; relative to the model group, ^*^ *P* < 0.05, ^**^ *P* < 0.01, ns: *P* > 0.05.

### 3.3 JGD regulates serum metabolite levels in smoke/LPS-exposed rats

To further characterize the metabolic alterations associated with smoke/LPS-induced pulmonary injury and JGD-H intervention, untargeted metabolomic profiling was performed. Principal component analysis (PCA) revealed clear separation among the groups (Figures 3A,D). Orthogonal partial least squares-discriminant analysis (OPLS-DA) was adopted to screen the information concerning sample classification. The model assessment values were R2Y = 0.999, Q2 = 0.830 (control-vs.-model) and R2Y = 0.991, Q2 = 0.878 (model-vs.-JGD-H), indicating good model fit and predictive ability (Figures 3B,E). In light of the permutation test (n = 200), all the simulated values fell short of the initial Q2 value on the right (Figures 3C,F). Using the predefined exploratory screening criteria, 114 candidate differentially abundant metabolites were shared between the two comparisons (Supplementary Figure S1; Figures 3G,H). KEGG analysis linked these metabolites mainly to carbon metabolism, cysteine and methionine metabolism, two-oxocarboxylic acid metabolism, taurine and hypotaurine metabolism, biosynthesis of amino acids, pyrimidine metabolism, arachidonic acid metabolism, and C5-branched dibasic acid metabolism (Figure 3I).

**FIGURE 3:**
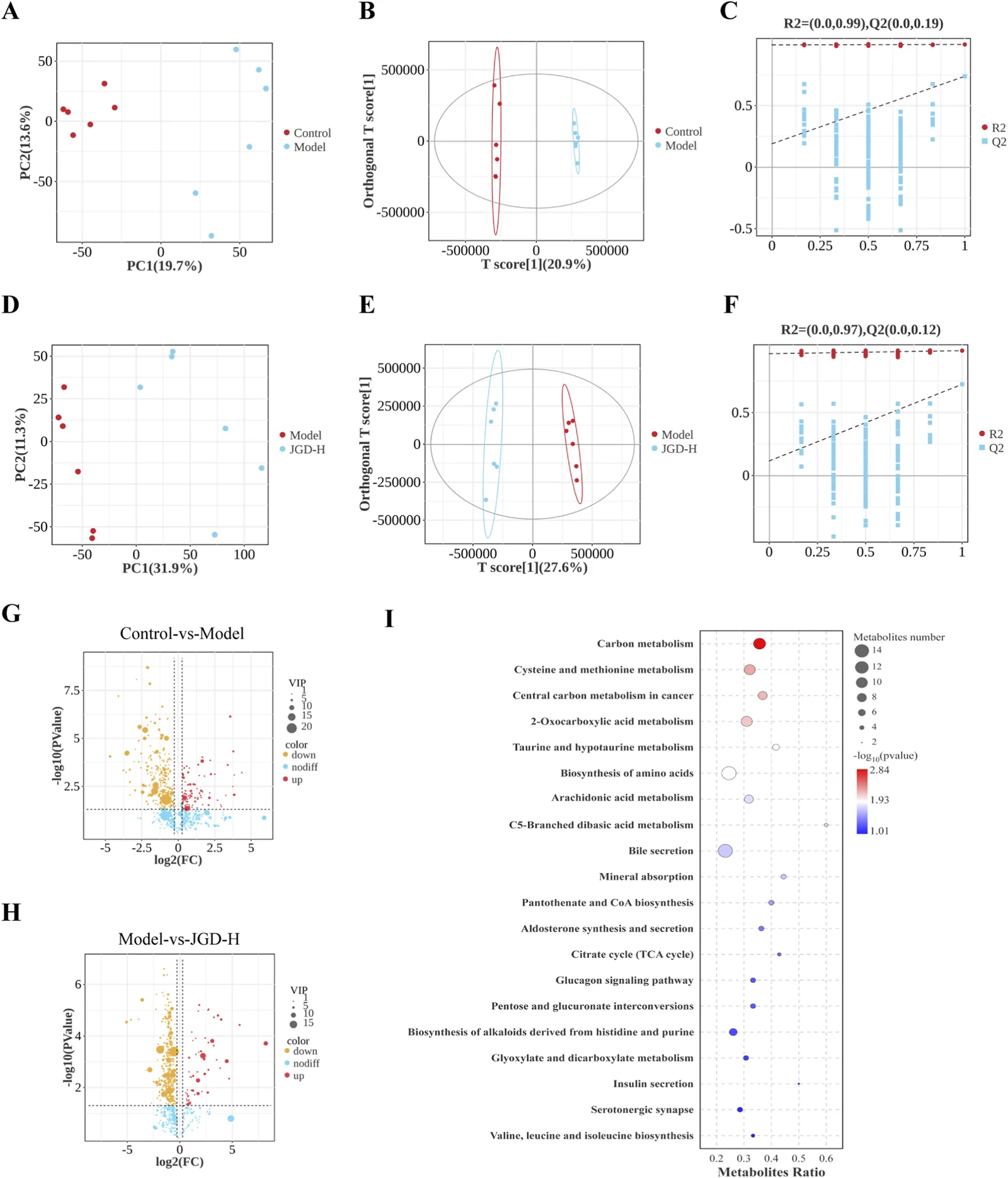
Serum metabolomics results of smoke/LPS-exposed rats after JGD treatment. **(A)** PCA score plot of the control and model groups. **(B)** OPLS-DA score plot between the control and model groups. **(C)** Permutation analysis between the control and model groups. **(D)** PCA score plot of the model and JGD-H groups. **(E)** OPLS-DA score plot between the model and JGD-H groups. **(F)** Permutation analysis between the model and JGD-H groups. **(G)** Volcano plot of candidate differentially abundant metabolites between the control and model groups. **(H)** Volcano plot of candidate differentially abundant metabolites between the model and JGD-H groups. **(I)** Bubble plot of the top 20 candidate metabolic pathways. n = 6 animals per group.

### 3.4 Integrated transcriptomic and metabolomic analyses suggest an association between the PPAR signaling pathway and arachidonic acid metabolism

To further clarify the molecular basis underlying the protective effects of JGD, an integrated analysis of the transcriptomic and metabolomic datasets was performed. As shown in Figure 4A, the top 10 coenriched pathways at both the gene and metabolite levels were identified, among which four pathways, namely, biosynthesis of amino acids, carbon metabolism, two-oxocarboxylic acid metabolism, and arachidonic acid metabolism, were significantly enriched. A heatmap further displays the expression patterns of the 44 candidate differentially abundant metabolites that were involved in the integrated analysis (Figure 4B), revealing distinct metabolic profiles among the control, model, and JGD-H groups. Given that arachidonic acid metabolism emerged as one of the most relevant shared pathways in our integrated analysis and considering the reported role of PPARγ in arachidonic acid metabolism and inflammatory responses in pulmonary disease (Wu et al., 2025; Wang et al., 2025), we next examined its association with the PPAR signaling pathway. The gene–metabolite interaction network revealed tight connections between the PPAR signaling pathway and arachidonic acid metabolites, with predominantly negative correlations observed between *Pparg* and 12 s-hydroxy-5z,8z,10e,14z-eicosatetraenoic acid (12 s-HETE); 5 s-hydroxy-6e,8z,11z,14z-eicosatetraenoic acid (5 s-HETE); leukotriene b4 (LTB4); arachidonic acid; and prostaglandin f2.alpha. (Figure 4C). We then assessed the relationships between the levels of arachidonic acid metabolites and pulmonary pathological and inflammatory indicators. Correlation analysis revealed that representative metabolites, including 12 s-HETE, 2,3-dinor-8-isoprostaglandin-f2.alpha, 5 s-HETE, LTB4, and arachidonic acid, were significantly positively correlated with these pathological indices (Figure 4D), suggesting that dysregulated arachidonic acid metabolism is closely associated with pulmonary inflammatory injury in smoke/LPS-exposed rats. To further characterize the arachidonic-acid-related metabolic pattern highlighted by the exploratory analysis, the relative abundances of representative metabolites were further examined. As shown in Figure 4E, compared with those in the control group, the relative abundances of LTB4, 5 s-HETE, and 12 s-HETE in the model group significantly increased, whereas those in the JGD-H group significantly decreased. Taken together, these findings suggest that the JGD-mediated alleviation of smoke/LPS-induced pulmonary inflammatory injury may be associated with the modulation of arachidonic acid metabolism, potentially through PPARγ-related signaling.

**FIGURE 4:**
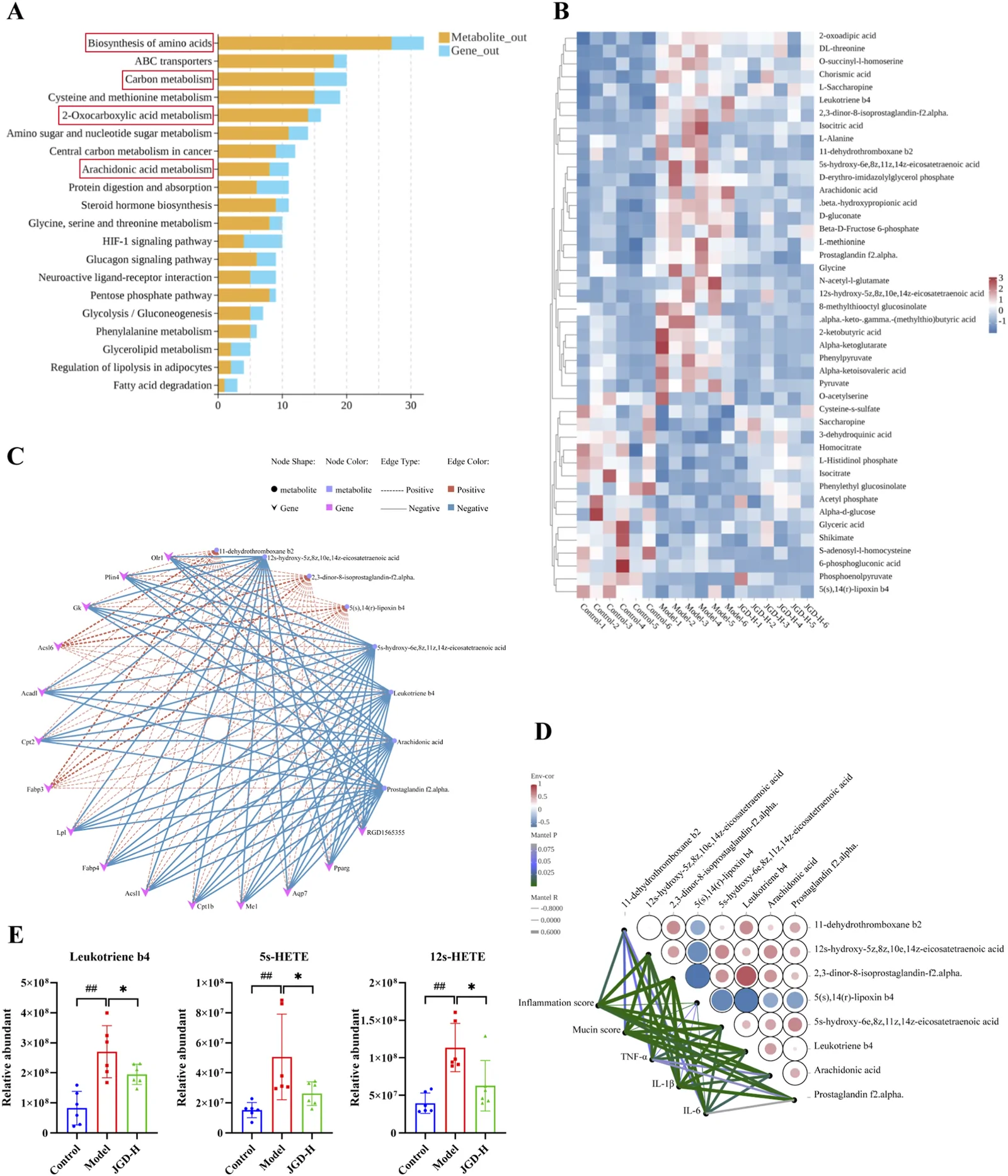
Integrated transcriptomic and metabolomic analyses associate the PPAR signaling pathway with arachidonic acid metabolism in smoke/LPS-exposed rats. **(A)** Top coenriched pathways shared by the transcriptomic and metabolomic datasets. **(B)** Heatmap showing the expressions of the differentially abundant metabolites involved in the integrated analysis among the control, model, and JGD-H groups. **(C)** Gene‒metabolite interaction network between the PPAR signaling pathway and arachidonic acid metabolites. Red and blue edges indicate positive and negative correlations, respectively. **(D)** Analysis of the correlations between the levels of arachidonic acid metabolites and pulmonary pathological and inflammatory indicators. **(E)** Relative abundances of leukotriene b4, 5 s-hydroxy-6e,8z,11z,14z-eicosatetraenoic acid (5 s-HETE), and 12 s-hydroxy-5z,8z,10e,14z-eicosatetraenoic acid (12 s-HETE) among the control, model, and JGD-H groups (n = 6 animals per group). Data are presented as the means ± SDs and were analyzed by one-way ANOVA followed by Tukey’s multiple-comparisons test **(E)**. Relative to the control group, ^##^ *P* < 0.01; relative to the model group, ^*^ *P* < 0.05.

### 3.5 JGD upregulates PPARγ expression *in vitro* and *in vivo*

Given that the exploratory integrated omics analysis prioritized PPARγ-related signaling as a candidate mechanism associated with the effects of JGD, we next examined PPARγ expression *in vivo* and *in vitro*. As shown in Figure 5A, Western blot analysis demonstrated that the PPARγ protein expressions in the lung tissues of the model rats markedly decreased and were partially restored after JGD treatment. Before the *in vitro* experiments were performed, the cytotoxicity of JGD toward RAW264.7 macrophages was evaluated by an MTT assay. As shown in Figure 5B, JGD at concentrations ranging from 1.5625 to 200 μg/mL did not significantly affect cell viability, whereas 400 μg/mL significantly reduced cell viability. Treatment with JGD (50, 100, or 200 μg/mL) strongly decreased LPS-induced inflammation in RAW264.7 cells in a dose-dependent manner (Supplementary Figure S2). Immunofluorescence staining revealed that LPS stimulation markedly decreased PPARγ fluorescence intensities in RAW264.7 cells, whereas JGD treatment restored PPARγ expressions in a concentration-dependent manner, with the most pronounced effect observed at 200 μg/mL (Figure 5C). Consistently, Western blot analysis indicated that JGD markedly upregulated PPARγ levels in LPS-induced RAW264.7 cells (Figure 5D). Collectively, these findings indicate that JGD restores PPARγ expressions both *in vivo* and *in vitro*, which may support the involvement of PPARγ in the protective effects of JGD against smoke/LPS-induced pulmonary inflammatory injury.

**FIGURE 5:**
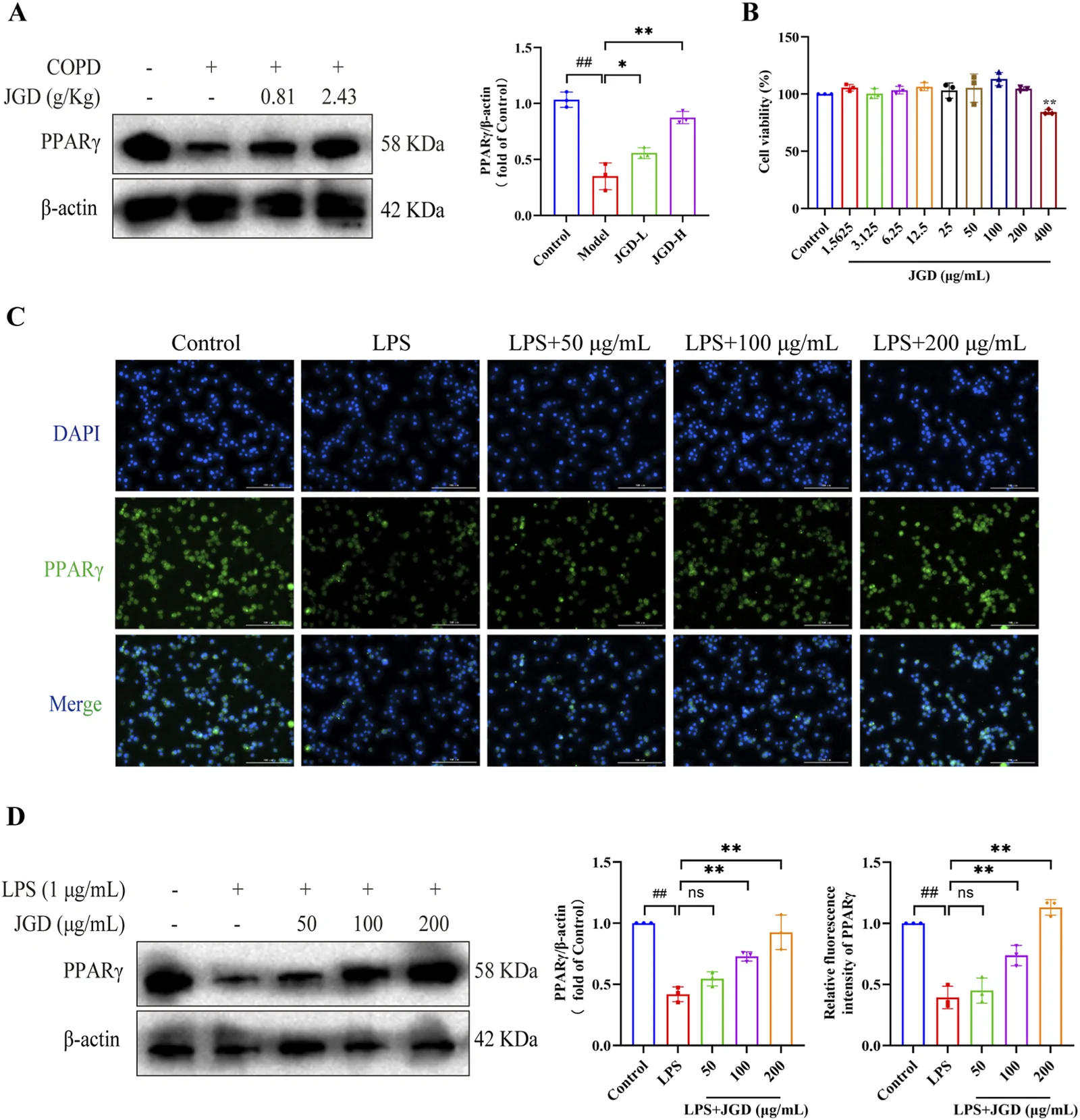
JGD increases PPARγ expressions both *in vitro* and *in vivo*. **(A)** Representative Western blot images and quantitative analysis of PPARγ expression in lung tissues from the indicated groups (n = 3 animals per group). **(B)** Viability of RAW264.7 cells treated with different concentrations of JGD (n = 6 independent experiments). **(C)** Representative immunofluorescence staining and quantification of PPARγ (green) and nuclear staining (DAPI, blue) in RAW264.7 cells (n = 3 independent experiments). Scale bars, 100 µm. **(D)** Representative Western blot images and quantitative analysis of PPARγ expression in RAW264.7 cells (n = 3 independent experiments). Data are presented as the means ± SDs and were analyzed by one-way ANOVA followed by Tukey’s multiple-comparisons test **(A–D)**. Relative to the control group, ^##^ *P* < 0.01; relative to the model group, ^*^ *P* < 0.05, ^**^ *P* < 0.01, ns: *P* > 0.05.

### 3.6 Chemical profiling of JGD and serum-detected prototype metabolites

A total of 156 JGD-derived metabolites, including 42 prenol lipids, 40 flavonoids, 31 isoflavonoids, and 13 organooxygen metabolites, were detected and annotated in JGD by UPLC‒MS/MS (Supplementary Table S1; Figures 6A,B). To characterize the absorbable JGD-derived metabolites *in vivo*, the base peak intensity chromatograms of the JGD extract and rat serum collected after JGD administration (Figure 6C) were analyzed and compared. A total of 77 prototype metabolites, including 28 prenol lipids, 22 flavonoids, and 13 isoflavonoids, were detected and annotated in serum (Figure 6D; Supplementary Table S2). To further explore the potential pharmacological basis of these absorbed prototype metabolites, a metabolite‒target interaction network was constructed using Cytoscape. The network contained 221 nodes, including 77 prototype metabolites and 144 predicted target genes (Figure 6E). The chemical structures of the absorbed prototype metabolites are shown in Figure 7.

**FIGURE 6:**
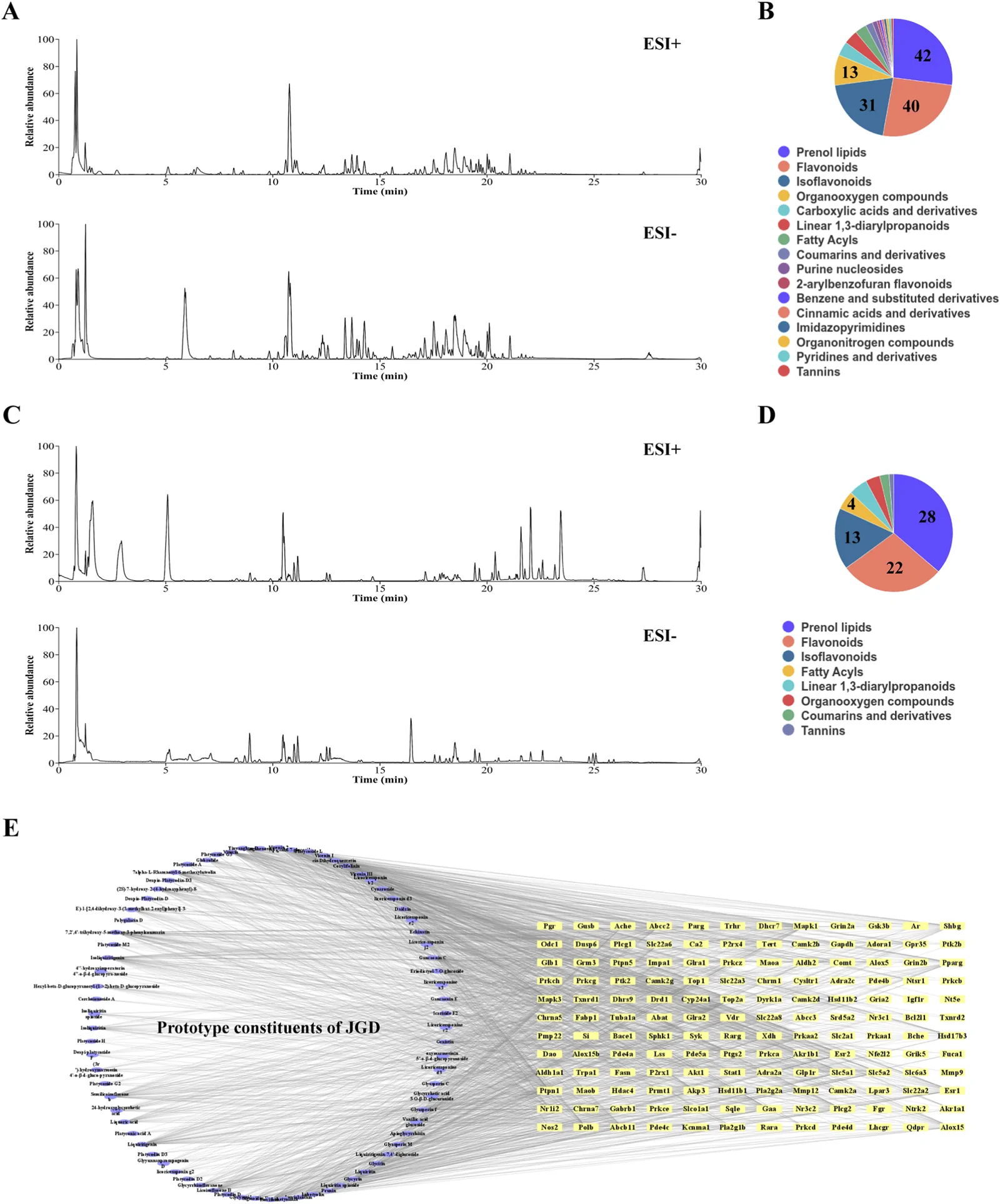
Chemical profiling of JGD and serum-detected JGD-derived metabolites by UPLC‒MS/MS. **(A)** Base peak intensity (BPI) of JGD *in vitro* in positive and negative patterns. **(B)** Structural categorization of the JGD-derived metabolites detected *in vitro*. **(C)** BPI of JGD *in vivo* in positive and negative modes. **(D)** Structural categorization of the absorbed prototype metabolites of JGD *in vivo*. **(E)** Network of the prototype metabolites of JGD in serum and the predicted target genes.

**FIGURE 7:**
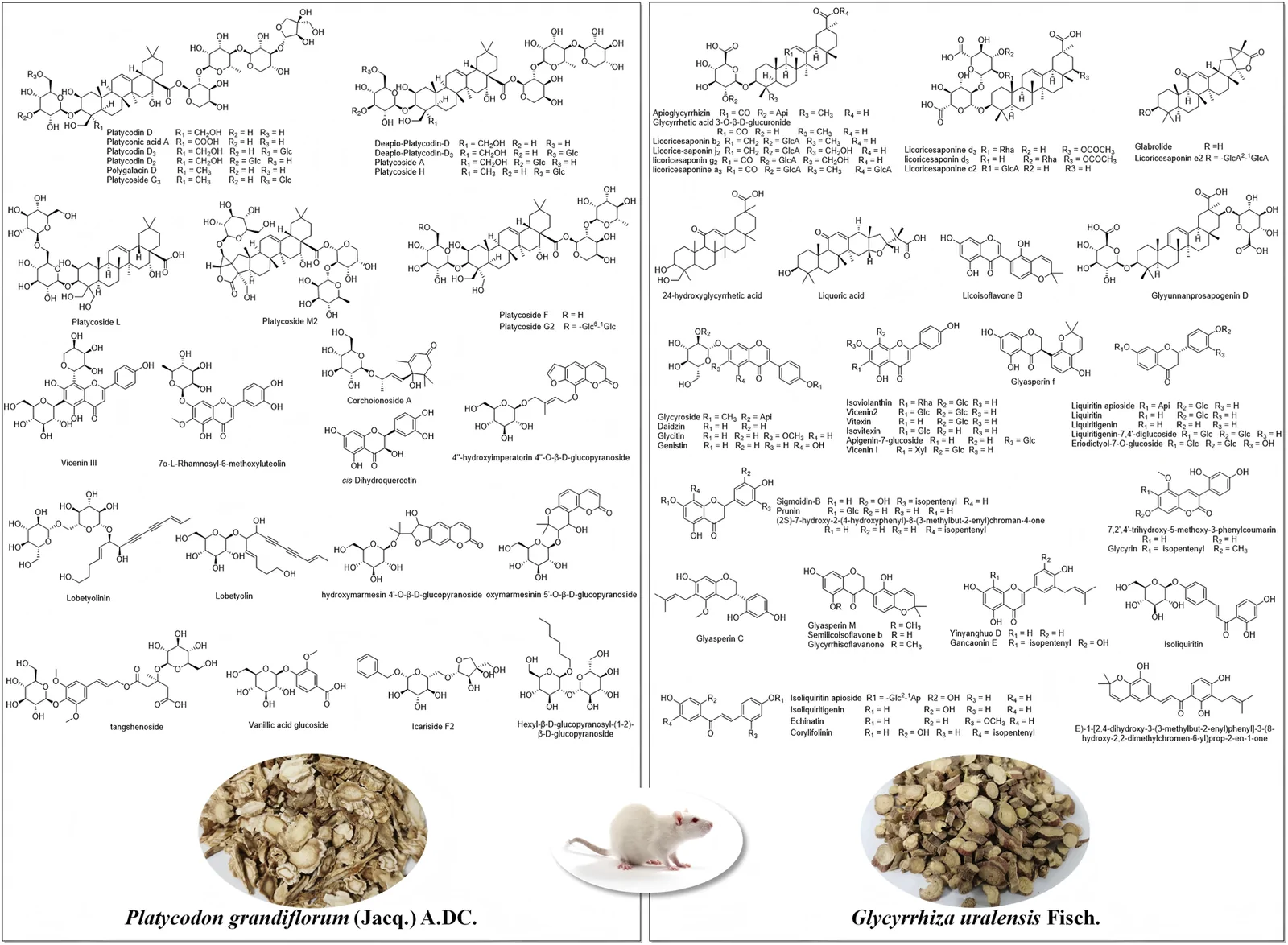
Chemical structures of JGD-derived prototype metabolites detected and annotated in serum.

### 3.7 LG attenuates inflammation in LPS-induced RAW264.7 cells in a PPARγ-associated manner

On the basis of the results of the preceding metabolite‒target interaction network analysis, we next aimed to identify potential bioactive metabolites in JGD that could directly interact with PPARγ. Molecular docking was employed to validate the binding affinities of the metabolites with PPARγ. A binding energy < −5.0 kcal/mol indicates that the molecule has excellent protein binding capacity (Chen et al., 2021). As shown in Table 1 and Figure 8A, cis-dihydroquercetin, LG, glyasperin f, echinatin, semilicoisoflavone b, and licoisoflavone B could bind tightly to PPARγ targets. On the basis of its relatively favorable docking performance and detection in serum, LG was selected for subsequent cell experiments. MTT assays revealed that LG at concentrations of 1.5625–12.5 μM did not significantly affect RAW264.7 cell viability (Figure 8B). Moreover, at concentrations of 3.125, 6.25, and 12.5 μM, LG decreased LPS-induced inflammatory cytokine levels in a concentration-dependent manner (Supplementary Figure S3). On this basis, 12.5 μM LG was used in the following mechanistic experiments. To determine whether LG exerted anti-inflammatory effects via PPARγ, RAW264.7 cells were stimulated with LPS in the presence or absence of LG and/or the selective PPARγ antagonist GW9662. As shown in Figure 8C, GW9662 further aggravated the inflammatory response, as evidenced by increased levels of TNF-α and IL-1β. Notably, cotreatment with LG partially attenuated the proinflammatory effect of GW9662. Immunofluorescence staining revealed that LPS markedly decreased PPARγ fluorescence intensities in RAW264.7 cells, whereas LG restored PPARγ expressions. In contrast, GW9662 further suppressed PPARγ fluorescence intensities, and this inhibitory effect was partially reversed by cotreatment with LG (Figure 8D). Western blot analysis revealed the same trend (Figure 8E). Because the exploratory omics analysis implicated arachidonic acid metabolism as a candidate pathway associated with JGD treatment, we further examined key enzymes involved in this pathway. The levels of cyclooxygenase-2 (COX-2) and 5-lipoxygenase (5-LOX) markedly increased after LPS stimulation and were further elevated by GW9662, whereas LG significantly reduced the levels of both enzymes under LPS stimulation and partially attenuated their increase in the presence of GW9662 (Figure 8F). Collectively, these findings indicate that LG suppresses LPS-induced inflammation in a PPARγ-associated manner and is associated with the modulation of downstream arachidonic acid metabolic enzymes.

**TABLE 1:** Binding energies of the serum-detected JGD-derived prototype metabolites binding to PPARγ.

No.	Prototype metabolite	Binding energy (kcal/mol)
1	24-Hydroxyglycyrrhetic acid	80.0186
2	7alpha-L-Rhamnosyl-6-methoxyluteolin	2.13514
3	cis-Dihydroquercetin	−27.7593
4	Cynaroside	−3.13481
5	Daidzin	1.50407
6	Echinatin	−11.8835
7	Gancaonin C	0.599621
8	Genistin	−1.09716
9	Glyasperin f	−16.4661
10	Glycyrrhetic acid 3-O-β-D-glucuronide	61.9832
11	Isoviolanthin	7.44048
12	Isovitexin	−3.19973
13	Licoisoflavone B	−9.98953
14	Licoricesaponin g2	70.7844
15	Liquiritigenin	−23.0581
16	Liquoric acid	84.0442
17	Semilicoisoflavone b	−10.4754

**FIGURE 8:**
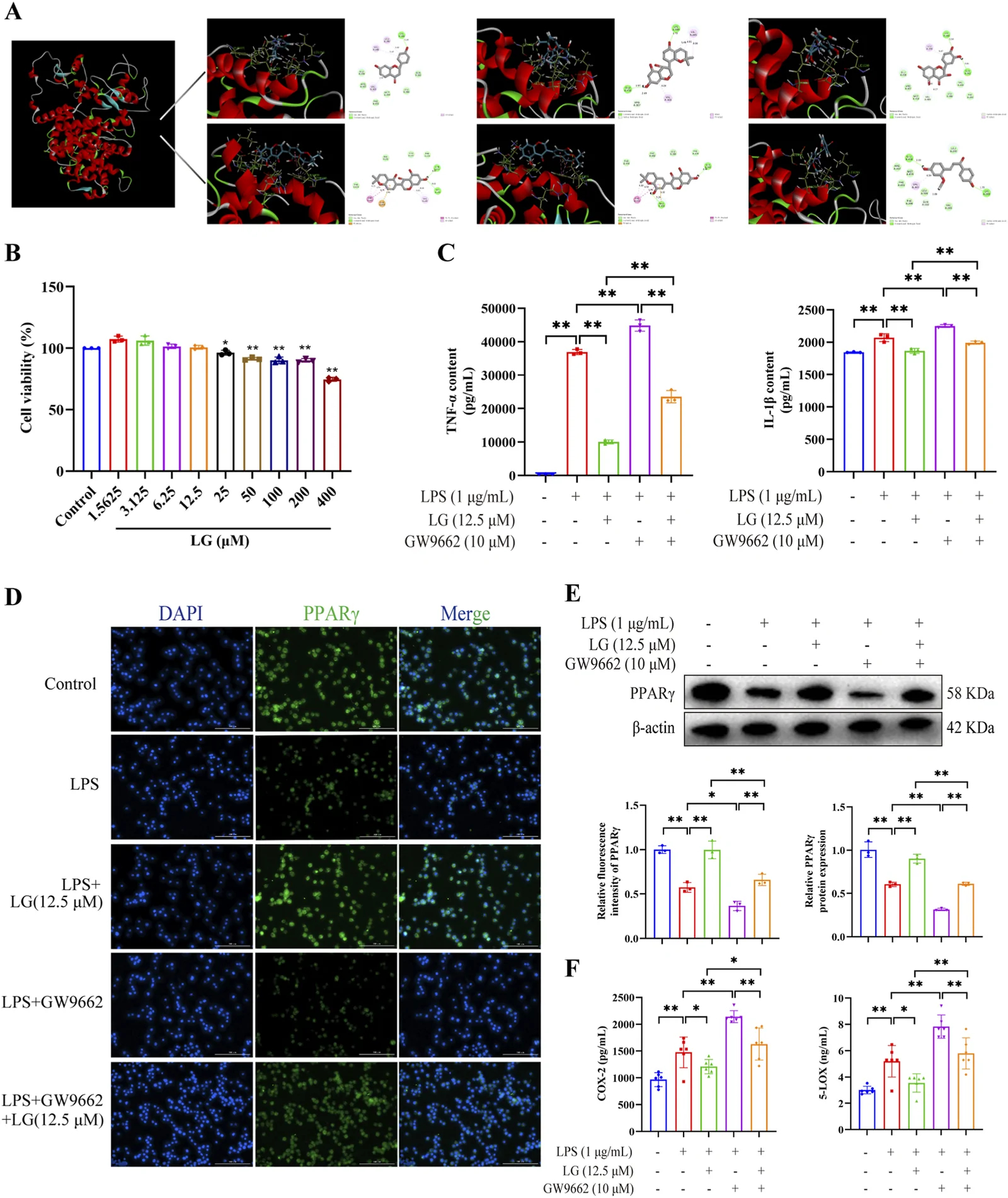
LG alleviates inflammation in LPS-induced RAW264.7 cells by increasing PPARγ expressions. **(A)** Molecular docking model of absorbed prototype metabolites (cis-dihydroquercetin, echinatin, glyasperin f, licoisoflavone B, liquiritigenin, and semilicoisoflavone **(B)** with PPARγ. **(B)** Viability of RAW264.7 cells treated with different concentrations of LG (n = 6 independent experiments). **(C)** Inflammatory cytokine (TNF-α and IL-1β) contents of RAW264.7 cells under the indicated treatments (n = 6 independent experiments). **(D)** Representative immunofluorescence staining and quantification of PPARγ (green) and nuclear staining (DAPI, blue) in RAW264.7 cells (n = 3 independent experiments). Scale bars, 100 µm. **(E)** Representative Western blot images and quantitative analysis of PPARγ expression in RAW264.7 cells (n = 3 independent experiments). **(F)** The levels of COX-2 and 5-LOX in RAW264.7 cells (n = 3 independent experiments). The data are presented as the means ± SDs and were analyzed by one-way ANOVA followed by Tukey’s multiple-comparisons test **(B–F)**. ^*^ *P* < 0.05, ^**^ *P* < 0.01.

### 3.8 JGD alleviates intestinal injury and restores barrier-associated protein expressions in smoke/LPS-exposed rats

To evaluate whether JGD could ameliorate intestinal alterations accompanying smoke/LPS-induced pulmonary injury, colonic histopathology, inflammatory cytokines, and barrier-related proteins were assessed. As shown in Figures 9A,E,H staining revealed that the model group exhibited marked histopathological damage, including mucosal structural disruption, inflammatory cell infiltration, and glandular disorder. Both the JGD-L treatment and the JGD-H treatment alleviated these pathological alterations, with the high-dose group showing a more evident protective effect. Consistent with the histological findings, the levels of proinflammatory cytokines in colon tissues were significantly greater in the model group than in the control group (Figure 9B). JGD treatment significantly reduced the levels of these cytokines. The MUC2 mucin and ZO-1 proteins play positive roles in sustaining the integrity of the intestinal barrier (He et al., 2023; Qiao et al., 2025). As shown in Figures 9C,D, smoke/LPS-exposed rats presented lower colonic MUC2 and ZO-1 expression than control rats did, whereas JGD-H markedly restored the expression of both proteins. Collectively, these findings indicate that JGD alleviates intestinal injury in smoke/LPS-exposed rats and preserves intestinal epithelial integrity.

**FIGURE 9:**
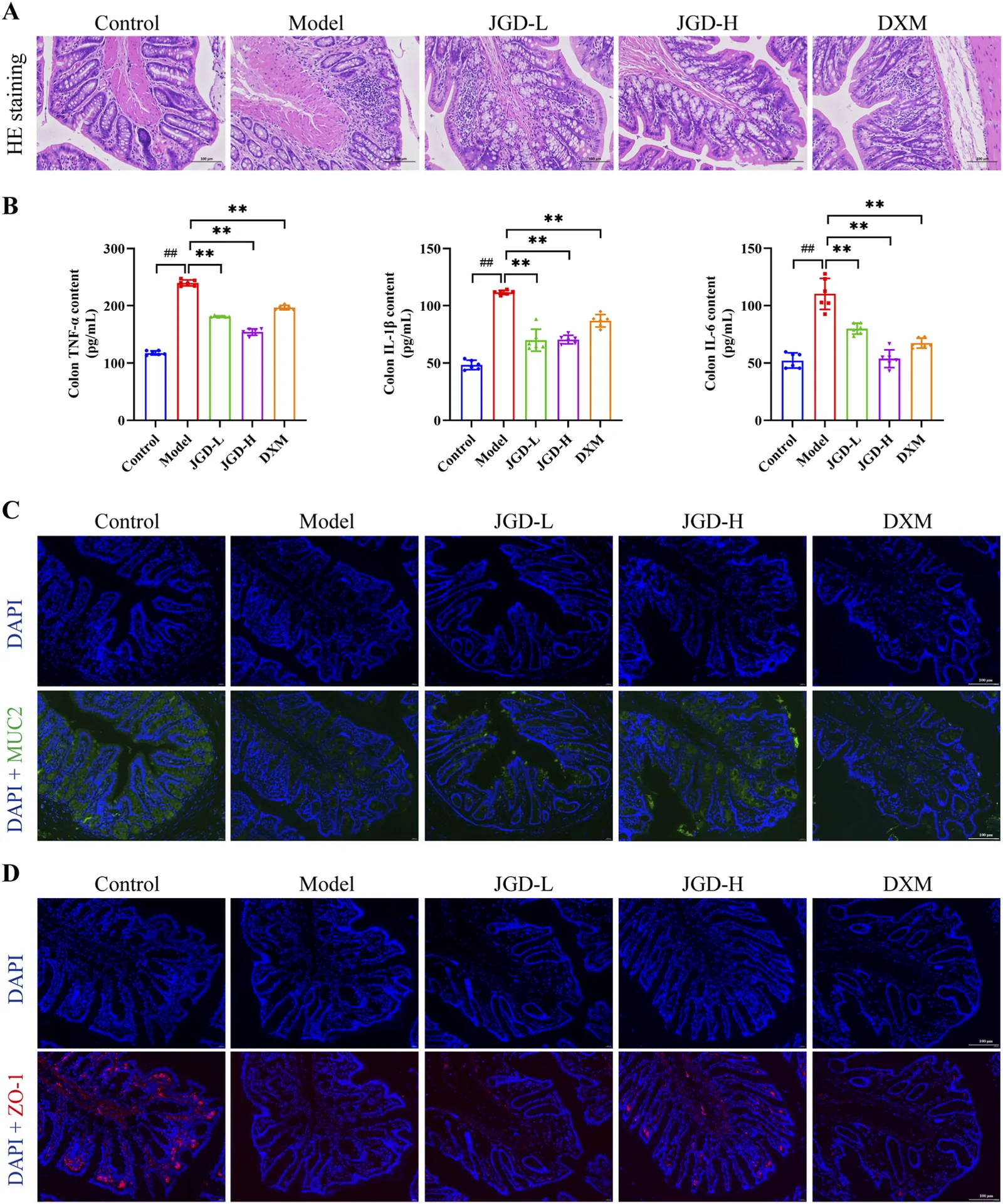
JGD alleviated intestinal injury and restored barrier-associated protein expressions in smoke/LPS-exposed rats. **(A)** Representative images of H&E-stained colon sections from the indicated groups (n = 3 animals per group). Scale bar: 100 μm. **(B)** Inflammatory cytokine contents in colon tissues (n = 6 animals per group). **(C)** Colon slices subjected to immunofluorescence staining for MUC2 (green) and nuclear staining (DAPI, blue) (n = 3 animals per group). Scale bar, 100 μm. **(D)** Colon slices were subjected to immunofluorescence staining for ZO-1 (red) and nuclear staining (DAPI, blue) (n = 3 animals per group). Scale bar, 100 μm. The data are presented as the mean ± SD and were analyzed by one-way ANOVA followed by Tukey’s multiple-comparisons test **(B)**. Relative to the control group, ^##^ *P* < 0.01; relative to the model group, ^**^ *P* < 0.01.

### 3.9 JGD alters the gut microbiota composition in smoke/LPS-exposed rats

16S rRNA sequencing was utilized to examine the influence of JGD on the gut microbiota. The rarefaction curves tended to be similar to those of the OTU, Sobs, and Shannon indices (Figure 10A), indicating that the sequencing data adequately represented the microbial diversity. As shown in Figure 10B, 527 OTUs were common among the groups. Alpha diversity analysis revealed that the Sobs index significantly differed among the groups (P = 0.0258), whereas the Shannon and Simpson indices did not significantly differ (P = 0.1744 and P = 0.4306, respectively) (Figure 10C). In contrast, the results of the principal coordinate analysis (PCoA) plot revealed an obvious separation in the beta diversity analysis, and the accumulation of the JGD group was closer to that of the control group (Figure 10D). This finding was further supported by ANOSIM, which revealed a significant difference between groups (R = 0.6955; P = 0.001) (Figure 10E), indicating that smoke/LPS exposure and JGD-H intervention were both associated with marked alterations in the microbial community structures. The overall structure of the gut microbiota was subsequently compared between bacterial taxa at the phylum and genus levels. At the phylum level, Firmicutes and Bacteroidetes accounted for the greatest percentages of the three groups (Figure 10F). Among these genera, *Lactobacillus*, *Bacteroides*, Lachnospiraceae_NK4A136_group, and Prevotellaceae_UCG-001 were the main genera in the three groups (Figure 10G). LEfSe analysis further revealed multiple discriminative taxa across the three groups (Figure 10H). Among these, representative taxa showing marked differences in distribution across groups included Bacilli, Lactobacillales, Lactobacillaceae, *Lactobacillus*, Akkermansia, *Akkermansia muciniphila*, Lachnospirales, Prevotellaceae, and Prevotellaceae_NK3B31_group (Figures 10H,I). Correlation analysis was performed between representative differential taxa, pulmonary pathological and inflammatory indicators, and arachidonic acid metabolites. As shown in Figure 10J, Lactobacillaceae, Akkermansia, and *A. muciniphila* were significantly negatively correlated with proinflammatory parameters and arachidonic acid metabolites. These findings suggest that JGD partially reshapes the gut microbiota composition in smoke/LPS-exposed rats and that changes in representative taxa are associated with host inflammatory phenotypes and arachidonic acid metabolites.

**FIGURE 10:**
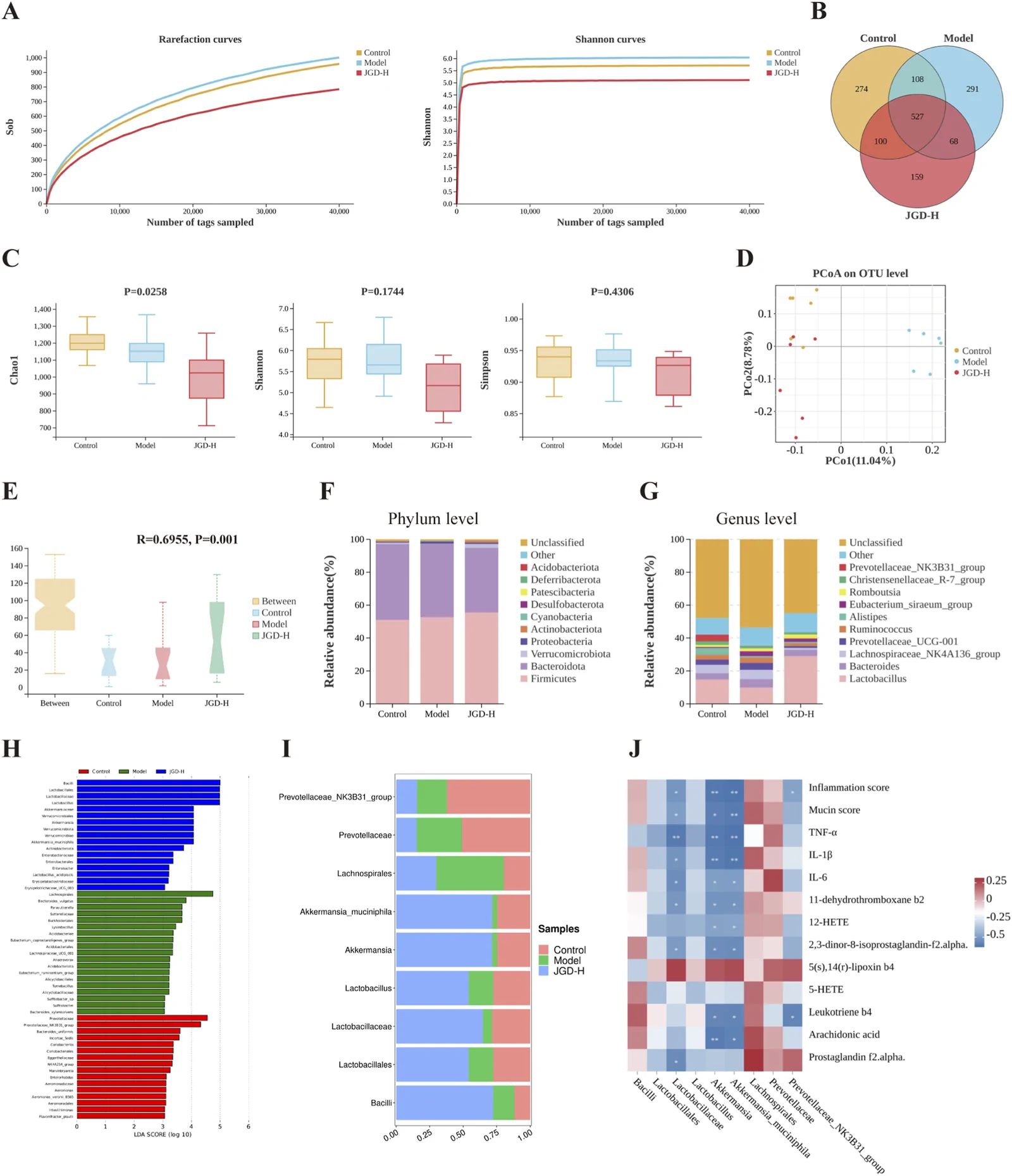
JGD reshaped the gut microbiota compositions in smoke/LPS-exposed rats. **(A)** Rarefaction curve used for determining Sobs and Shannon indicators at the OTU level (n = 6 animals per group). **(B)** Venn diagram of the OTUs in the indicated groups. **(C)** Chao1, Shannon, and Simpson alpha diversity indices at the OTU level. **(D)** PCoA among the control, model and JGD-H groups on the basis of unweighted UniFrac distances. **(E)** ANOSIM among the control, model and JGD-H groups. **(F)** Stacked bar plot of species in the indicated groups at the phylum level. **(G)** Stacked bar plot of the species in the indicated groups at the genus level. **(H)** LEfSe analysis of the comparative abundance of the gut microbiota among the control, model and JGD-H groups. **(I)** Relative distributions of representative differential taxa among the control, model, and JGD-H groups. **(J)** Analysis of correlations between representative differential taxa, arachidonic acid metabolites and pulmonary pathological and inflammatory indicators.

## 4 Discussion

COPD remains a major global public health challenge, affecting more than 400 million people worldwide and continuing to be the third leading cause of death globally. The estimated prevalence among adults aged 40 years and older was 10.6% in 2020, corresponding to approximately 480 million cases, and its burden is expected to continue rising (Lin et al., 2023). In this context, the development of safer and more effective therapeutic strategies for treating COPD is urgently needed. TCM formulas, with their multimetabolite and multitarget characteristics, provide a promising strategy for intervening in complex pathological networks (Cao et al., 2026). Our findings revealed that JGD alleviated pulmonary histopathological damage and was concurrently associated with improvements in intestinal pathological injury, the restoration of MUC2 and ZO-1 expressions, and alterations in the gut microbial composition. These intestinal and microbial changes occurred alongside reduced pulmonary inflammation and alterations in arachidonic acid metabolism. These results extend the pharmacological understanding of JGD beyond its traditional lung-related application and suggest that intestinal protection may represent an important but previously overlooked feature of this classic formula. In addition, chemical profiling and serum pharmacochemistry analysis enabled the detection and annotation of JGD-derived prototype metabolites in serum, from which LG was further identified as a candidate bioactive metabolite contributing to the PPARγ-mediated anti-inflammatory effects of JGD.

Emerging evidence underscores the pivotal role of chronic systemic inflammation in the pathogenesis of COPD, wherein persistent airway inflammation drives a self-perpetuating cycle of tissue damage and immune dysregulation (Barnes, 2016; Keely et al., 2012). In the present study, transcriptomic analysis revealed the PPAR signaling pathway as one of the most responsive pathways after JGD treatment, and the affected genes were distributed mainly in the PPARγ-associated branch, including *Fat/Cd36*, *Cpt-1*, *Cpt-2*, *Lcad*, and *aP2*. These molecules are closely involved in lipid uptake, fatty acid transport, and β-oxidation (Bao et al., 2025; Guo et al., 2023). The downregulation of these genes in the model group suggested that smoke/LPS exposure was accompanied by impaired lipid metabolic homeostasis in the lung, whereas JGD restored this metabolic program. PPARγ is a ligand-activated nuclear receptor with well-established roles in anti-inflammatory regulation and lipid metabolism (Marques et al., 2023). In pulmonary macrophage-driven inflammatory responses, reduced PPARγ activities are generally associated with excessive inflammatory signaling, whereas the restoration of PPARγ tends to restrain macrophage activation and promote the resolution of lung inflammation (Wang J. et al., 2024). Therefore, the recovery of PPARγ and its downstream targets observed here provides correlational evidence that is consistent with a mechanistic role for PPARγ in the reduction in inflammatory injury observed after JGD treatment. Notably, our findings support the findings of a previous report that JGD protected against bleomycin-induced pulmonary fibrosis mainly by modulating phosphatidylinositol-related lipid changes and inhibiting the apoptosis of type II alveolar epithelial cells via the PI3K/Akt axis (Xu et al., 2022). By comparison, the results of the present COPD-like pulmonary injury study suggest that JGD acts through a distinct but related regulatory pattern in which the predominant molecular signature involves the recovery of PPARγ-associated genes that govern lipid handling.

COPD is increasingly recognized as a disease accompanied by profound metabolic reprogramming, particularly in lipid metabolism (Liang et al., 2025; Xie et al., 2025). Notably, the differentially abundant metabolites that were identified by untargeted metabolomics include both pharmacologically relevant secondary metabolites and primary metabolites that likely reflect general metabolic disturbances that are associated with the COPD phenotype rather than specific pharmacological targets of JGD. A mechanistic discussion focused on arachidonic acid metabolites with established roles in inflammatory signaling. Among the altered lipid pathways reported in previous studies, arachidonic acid metabolism has been among the most consistently implicated pathways and is closely linked to inflammatory responses in COPD (Du et al., 2025). Integrated transcriptomic and metabolomic analyses consistently revealed this pathway, and several representative metabolites, including 12 s-HETE, 5 s-HETE, and LTB4, were markedly increased in COPD rats but decreased after JGD. Importantly, these metabolites were positively correlated with the inflammatory score, mucus score, and proinflammatory cytokines, indicating that aberrant arachidonic acid metabolism was strongly linked to smoke/LPS-associated pulmonary inflammatory injury in our model. Arachidonic acid metabolic dysregulation is closely linked to inflammatory responses in COPD because excessive generation of proinflammatory eicosanoids, such as 12-HETE, 15 s-HETE, 11-HETE, PGE2, and TXB2, can amplify inflammatory injury by promoting inflammatory cell infiltration, cytokine release, oxidative stress, and airway remodeling, thereby accelerating disease progression (Huang et al., 2025). Mechanistically, PPARγ may regulate arachidonic acid metabolism by suppressing key proinflammatory enzymes, such as COX-2 and 5-LOX, and thereby limiting the generation of downstream eicosanoids, including LTC4 and PGD2 (Wu et al., 2025). Notably, the negative correlations among PPAR-related genes and arachidonic acid metabolites indicate an association between PPARγ-related signaling and arachidonic acid metabolism following JGD treatment. The combined omics and validation data are consistent with the possibility that the PPARγ–arachidonic acid metabolism axis may represent an important pathway associated with the protective effects of JGD against COPD-like pulmonary injury.

Given the central role of PPARγ restoration in the protective effects of JGD against smoke/LPS-induced pulmonary injury, identifying the JGD-derived metabolites responsible for this activity is of particular mechanistic relevance. Molecular docking suggested that several JGD-derived metabolites, including cis-dihydroquercetin, LG, glyasperin F, echinatin, semilicoisoflavone B, and licoisoflavone B, may interact with PPARγ. Among these candidates, LG was selected as a representative serum-detected JGD-derived metabolite for further experimental evaluation because it showed a relatively favorable docking result. This interpretation is further supported by previous studies. LG has been reported to possess anti-inflammatory and cytoprotective properties in various experimental models, including protection against lung cell injury through the inhibition of bacterial toxin production (Dai et al., 2013) and the alleviation of organ damage in renal injury models (Guo et al., 2024). While these studies were conducted in non-COPD settings, they support the broader pharmacological potential of LG as an anti-inflammatory agent. In the present study, LG increased PPARγ expression and attenuated the inflammatory response in LPS-stimulated RAW264.7 cells, whereas these effects were markedly blunted by pharmacological inhibition of PPARγ, supporting the view that LG exerts its activity at least partially through PPARγ-related signaling *in vitro*.

An important methodological consideration concerns the exploratory nature of the metabolite–target network shown in Figure 6E. As highlighted by Diao et al. (2026), database-driven network pharmacology analyses of complex herbal formulations are subject to inherent limitations, including database-related biases, false-positive predictions, and nonspecific compound–target associations. Accordingly, computationally predicted interactions should not be interpreted as definitive evidence of pharmacological activity or target specificity. In the present study, to improve the biological relevance of candidate selection, we restricted the network analysis to 77 JGD-derived prototype metabolites experimentally detected in rat serum following oral administration, rather than relying solely on constituents identified in the herbal extract. Nevertheless, serum detection alone does not establish pharmacological activity or specific target engagement. Therefore, the network analysis was used primarily as a hypothesis-generating approach, and the proposed mechanisms were further investigated through integrated transcriptomic and metabolomic analyses and subsequent experimental studies. The observed effects of JGD and liquiritigenin on PPARγ expression, together with the findings from GW9662-treated macrophages, provide evidence consistent with the involvement of PPARγ-related signaling. However, these findings do not establish direct binding between liquiritigenin and PPARγ or validate the other predicted compound–target interactions. Further studies involving direct target-engagement assays and quantitative pharmacokinetic assessments are warranted to clarify these potential interactions.

In addition to the lung, JGD treatment was accompanied by pathological intestinal and gut microbial alterations, providing a basis for further investigation of potential gut–lung interactions. In the present study, smoke/LPS-exposed rats exhibited evident pathological colonic injury, increased inflammatory cytokine levels, and reduced expressions of MUC2 and ZO-1, indicating disruption of intestinal mucosal integrity. JGD markedly ameliorated these abnormalities, suggesting that its protective effects were not restricted to the respiratory tract. These findings are mechanistically relevant because the intestinal epithelial barrier, which is composed mainly of epithelial cells and tight junctions, is essential for intestinal defense, and its disruption leads to increased permeability, bacterial translocation, inflammatory activation, and intestinal dysfunction (Gustafsson and Johansson, 2022; Tao et al., 2025). Recent evidence has consistently shown that the preservation of intestinal barrier integrity in patients with COPD not only reduces intestinal inflammatory cytokine levels but also alleviates pulmonary dysfunction and histopathological injury, accompanied by reduced pulmonary IL-1β, IL-6, and TNF-α levels (Liu et al., 2025; Wang et al., 2026). Furthermore, 16S rRNA sequencing demonstrated that smoke/LPS exposure was associated with a marked shift in microbial community structure, whereas JGD treatment partially normalized this pattern. As the gut microbiota is a key regulator of mucosal immunity and metabolic homeostasis, its dysbiosis in COPD may aggravate inflammation and impair immune function (Bowerman et al., 2020; Wang et al., 2023). Notably, representative taxa such as Lactobacillaceae and Akkermansia were negatively associated with inflammatory phenotypes and proinflammatory arachidonic acid metabolites in our study. The abundance of *Lactobacillus*, which is known to participate in the regulation of inflammatory immune responses (Mortaz et al., 2013), markedly decreased in smoke/LPS-exposed rats but increased after JGD treatment. These observations are consistent with previous evidence that *Lactobacillus* supplementation can attenuate emphysematous changes and reduce inflammatory cytokine levels in COPD model lungs (Shen et al., 2024). Similarly, Akkermansia has been recognized as a beneficial bacterium that is capable of reducing inflammation and reinforcing gut barrier function, thereby contributing to protection against inflammation-associated disorders (Cortijo-Alfonso et al., 2025; Wang et al., 2024b). Taken together, these findings suggest that JGD may alleviate COPD-like pulmonary inflammation in association with preserved intestinal barrier integrity and reshaped gut microbial homeostasis, although the causal contribution of these intestinal changes to pulmonary protection has not been established through interventional approaches such as fecal microbiota transplantation.

In summary, JGD attenuated pulmonary inflammatory and pathological injury in association with changes in PPARγ expression and arachidonic acid metabolism. JGD treatment was also accompanied by improvements to intestinal pathological injury, restoration of MUC2 and ZO-1 expressions, and alterations in the gut microbial composition. However, several limitations should be acknowledged. First, we acknowledge that the high dose of JGD used in this study (2.43 g/kg/day) exceeds the 1 g/kg/day threshold that is recommended by the journal consensus guidelines. Although previous experimental research using the same JGD formulation reported anti-inflammatory effects in rats at nominal doses of 0.84 and 3.36 g/kg (Huang et al., 2024), differences in disease models, extract preparation, and treatment duration limit direct comparisons between the studies. Second, the present study did not include direct pulmonary function measurements, such as the FEV0.1/FVC, airway resistance, or dynamic lung compliance. Therefore, the present findings should be interpreted in the context of COPD-like pulmonary injury. Third, intestinal barrier integrity was assessed using only MUC2 and ZO-1 immunofluorescence; a more comprehensive characterization incorporating additional tight junction proteins and functional permeability assays is warranted. Fourth, the associations among the gut microbiota, arachidonic acid metabolism, and pulmonary protection were inferred mainly from correlation analysis; mechanistic approaches such as fecal microbiota transplantation or targeted microbial intervention are needed to establish causality. Finally, although our experimental findings support the involvement of PPARγ-related signaling in the anti-inflammatory effects of JGD and identify LG as a candidate bioactive metabolite, direct target engagement and the contributions of other absorbed JGD-derived metabolites remain to be established. Future studies employing conditional PPARγ knockout models, direct assessments of pulmonary function and airflow limitation, functional intestinal permeability assays, and alternative microbiota approaches are warranted to address these limitations.

## 5 Conclusion

JGD is a traditional Chinese medicinal formulation with a history dating back approximately 1,826 years. Clinically, JGD has been utilized as a foundational prescription in the management of various pulmonary disorders. Previous research has predominantly concentrated on the addition or subtraction prescriptions of JGD, neglecting to investigate the fundamental formulations comprising the two botanical drugs *P. grandiflorus* and *G. uralensis*. This study is the first to investigate the therapeutic mechanisms and identify the principal bioactive metabolites of JGD in the treatment of COPD-like pulmonary injury. These findings demonstrate that JGD significantly alleviates pulmonary and intestinal injury in smoke/LPS-exposed rats. The underlying mechanism may involve the upregulation of PPARγ expression, which is associated with the modulation of arachidonic acid metabolism. In parallel, JGD treatment was accompanied by the restoration of intestinal barrier-associated protein expression and alterations in the gut microbial composition, which correlated with reduced levels of arachidonic acid-related metabolites, suggesting a potential contributory role in attenuating inflammatory responses in COPD-like pulmonary injury. This research provides integrative multiomics and experimental evidence supporting a potential lung-gut protective mechanism of JGD and suggests that JGD, along with its bioactive metabolite LG, holds promise for further development as a therapeutic intervention for COPD-like pulmonary injury.

## Data Availability

The data presented in this study are deposited in the Genome Sequence Archive (GSA) at the National Genomics Data Center, China National Center for Bioinformation / Beijing Institute of Genomics, Chinese Academy of Sciences (https://ngdc.cncb.ac.cn/gsa), under BioProject accession number PRJCA073530 and GSA accession number CRA049507. Additional supporting data are provided in the Supplementary Material.
