# Supplementary material for: Jiegeng decoction ameliorates COPD-like pulmonary injury in association with changes in PPARγ expression, arachidonic acid metabolism, and gut microbial composition
Source: Front Pharmacol. 2026 Oct 28;17:1933011. doi: 10.3389/fphar.2026.1933011 (PMC13751962; doi:10.3389/fphar.2026.1933011)
Supplement: Supplementary file 1 [file Supplementaryfile1.docx]

Supplementary Material

**1. Preparation of test samples**

Five batches of *Platycodon grandiflorus* (Jacq.) A. DC. and *Glycyrrhiza uralensis* Fisch. samples were pulverized separately and passed through a 60-mesh sieve. Precisely weighed powders of *P. grandiflorus* (1.00 g) and *G. uralensis* (1.00 g) were thoroughly mixed at a ratio of 1:2 (w/w) to prepare five sample combinations, designated as **Group 1** (*P. grandiflorus*, batch No. 202607016; *G. uralensis*, batch No. 20260701), **Group 2** (*P. grandiflorus*, batch No. 202604160; *G. uralensis*, batch No. 20260503), **Group 3** (*P. grandiflorus*, batch No. 202605262; *G. uralensis*, batch No. 20260604), **Group 4** (*P. grandiflorus*, batch No. 202607122; *G. uralensis*, batch No. 20260603), and **Group 5** (*P. grandiflorus*, batch No. 202607015; *G. uralensis*, batch No. 20260512). Each sample was decocted with 40 mL of water for 30 min and filtered. The residue was then decocted again with 30 mL of water for 30 min. The two filtrates were combined and transferred to a 100-mL volumetric flask. After cooling to room temperature, the solution was diluted to volume with water and thoroughly mixed. All five batches were prepared using the same procedure. An aliquot (1 mL) of each sample solution was withdrawn, passed through a membrane filter, and subjected to HPLC analysis for quantification of liquiritin, platycodin D, and glycyrrhizic acid.

**2. Preparation of reference standard solutions**

Approximately 1.00 mg each of platycodin D, glycyrrhizic acid, and liquiritin reference standards, previously dried to constant weight, was accurately weighed and dissolved separately in 50% ethanol in 10-mL volumetric flasks to obtain individual working solutions at approximately 0.100 mg/mL. Aliquots of 0.10, 0.30, 0.50, 0.70, 1.00, 1.40, and 1.80 mL of each working solution were accurately transferred into 10-mL volumetric flasks and diluted to volume with 50% ethanol. The resulting standard solutions were analyzed under the established HPLC conditions, and the corresponding peak areas were recorded. Calibration curves for liquiritin, glycyrrhizic acid, and platycodin D were constructed by linear regression using concentration as the independent variable and peak area as the dependent variable.

**3. HPLC conditions**

HPLC analysis was performed using a Waters e2695 HPLC system (Waters, Shanghai, China) equipped with a YMC-Pack ODS-A column (250 × 20 mm, 5 μm, 12 nm; YMC Co., Ltd., Japan). The column temperature was maintained at 40 °C. The mobile phase consisted of HPLC-grade acetonitrile (A) and 0.1% trifluoroacetic acid aqueous solution (B). Detection was performed at 276 nm with an injection volume of 30 μL and a flow rate of 1.0 mL/min. Gradient elution was conducted by increasing the proportion of solvent A from 5% to 50%.

**4. Determination and consistency evaluation of three marker compounds in five sample batches**

Using the above method, the calibration equations were obtained as follows: platycodin D, y=199820832x−110538 (R²=0.9994); glycyrrhizic acid, y=747870379x − 501757 (R² = 0.9999); and liquiritin, y=1608593200x−570346.4 (R² = 0.9991).

The calculated contents of liquiritin (**compound 1**), platycodin D (**compound 2**), and glycyrrhizic acid (**compound 3**) are summarized in **Table S1**, while the HPLC chromatograms of the five sample batches are shown in **Figure S1**.

**Table S1.** Contents of three marker compounds in five sample batches

| **Samples** | **Peak area of Comp-1** | **The contents of Comp-1** | **Peak area of Comp-2** | **The contents of Comp-2** | **Peak area of Comp-3** | **The contents of Comp-3** |
| --- | --- | --- | --- | --- | --- | --- |
| **Group 1** | 2826116 | 0.6890% | 5417687 | 2.5827% | 170343 | 0.4589% |
| **Group 2** | 2551877 | 0.6343% | 5326104 | 2.5466% | 170043 | 0.4682% |
| **Group 3** | 2759815 | 0.6741% | 5371721 | 2.5573% | 169361 | 0.4671% |
| **Group 4** | 2814674 | 0.6874% | 5479847 | 2.6125% | 170786 | 0.4598% |
| **Group 5** | 2787169 | 0.6814% | 5319130 | 2.5410% | 171079 | 0.4703% |

As shown in Table 1, the contents of liquiritin, platycodin D, and glycyrrhizic acid were comparable among the five sample batches prepared using the same extraction procedure, indicating satisfactory batch-to-batch consistency of the herbal materials.

**Figure S1.** The HPLC chromatograms of the five sample batches

**5. Similarity analysis of HPLC fingerprints of five sample batches**

In addition to the three marker compounds described above, the overall chromatographic profiles of the five batches were further evaluated for similarity. Chromatographic peaks were matched according to their retention times and elution order, and peaks occurring at comparable retention times with consistent elution sequences across all five batches were defined as common peaks and designated P1–P10. To assess the reliability of peak matching, the mean retention time, standard deviation (SD), and relative standard deviation (RSD) of each common peak were calculated across the five batches according to: RSD(%) =$\frac{SD}{RT}\times100\%$.The RSD values of the retention times for the 10 common peaks ranged from 0.0547% to 0.2607%, indicating minimal retention-time variation among batches and supporting the reliable assignment of P1–P10.

**Table S2.** Chromatographic information of the 10 common peaks

| Common peak No. | Mean RT (min) | RT-RSD/% | Mean reference peak area |
| --- | --- | --- | --- |
| P1 | 10.2386 | 0.2607 | 236521.4 |
| P2 | 10.9786 | 0.2096 | 375321.6 |
| P3 | 11.5942 | 0.2478 | 327359.0 |
| P4 | 13.1244 | 0.1432 | 236332.6 |
| P5 | 13.4148 | 0.1394 | 2176095.4 |
| P6 | 13.7840 | 0.1386 | 2723643.4 |
| P7 | 15.3692 | 0.1199 | 271561.6 |
| P8 | 17.3426 | 0.1053 | 50521.6 |
| P9 | 18.5760 | 0.0855 | 373905.0 |
| P10 | 22.4546 | 0.0547 | 722355.0 |

On this basis, the similarity between each sample and the mean reference fingerprint was evaluated using cosine similarity, with the peak areas of the common peaks as fingerprint variables. For the (j)^th^ common peak, the arithmetic mean of the corresponding peak areas from the five samples was used to construct the mean reference fingerprint: Rⱼ =$\frac{1}{5}\sum_{i=1}^{5} A_{ij}$, where $A_{ij}$ represents the peak area of the (j)^th^ common peak in the (i)th sample, and Rⱼ represents the corresponding peak area in the mean reference fingerprint.

The similarity between each sample and the mean reference fingerprint was then calculated using cosine similarity. For sample (i), the peak areas of the 10 common peaks were expressed as the vector X𝑖=(A_𝑖1_,A_𝑖2_,…,A_𝑖10_), The reference fingerprint vector, R=(R₁,R₂,…,R₁₀) was constructed from the 10 mean peak-area values Rⱼ. The similarity was calculated as Sᵢ = $\frac{\sum_{j=1}^{10} A_{ij}R_{j}}{\sqrt{\sum_{J=1}^{10} A^{2}ⅈj}\sqrt{\sum_{j=1}^{10} R_{j}^{2}}}$. A cosine similarity value closer to 1 indicates a greater similarity in the relative distribution of common-peak areas and, consequently, a higher overall similarity between chromatographic fingerprints.

Consequently, the five batches showed high similarity to the mean reference fingerprint, with similarity values of 0.9997, 0.9989, 0.9977, 0.9963, and 0.9873, respectively, and a mean value of 0.9960, indicating strong consistency in their overall chemical profiles and common-peak distribution patterns. Pairwise similarity was highest between samples 3 and 4 (0.9996) and lowest between samples 4 and 5 (0.9707). Overall, the high retention-time reproducibility and peak-area similarity confirmed stable chromatographic profiles across the five batches, although sample 5 showed moderate variation in the relative areas of several common peaks.
