# Supplementary material for: Jiegeng decoction ameliorates COPD-like pulmonary injury in association with changes in PPARγ expression, arachidonic acid metabolism, and gut microbial composition
Source: Front Pharmacol. 2026 Oct 28;17:1933011. doi: 10.3389/fphar.2026.1933011 (PMC13751962; doi:10.3389/fphar.2026.1933011)
Supplement: Supplementary file 2 [file Supplementaryfile2.docx]

Supplementary Material

# 1 Supplementary Methods

**1.1 Chemical characterization of JGD**

1.0 g of JGD sample and 40 mL 80% methanol were charged into a 50 mL centrifuge tube, and ultrasonicated for 30 minutes. After that, 1 mL suspension was transferred into a 1.5 mL tube and centrifuged at 4 °C and 12000 rpm for 10 min. Finally, 100 μL of supernatant was mixed with 100 μL ultrapure water and then pipetted into an injection vial for detection.

A Vanquish Flex UHPLC chromatograph (Thermo Fisher Scientific, Inc., Waltham, MA, USA) equipped with an ACQUITY UPLC HSS T3 column (2.1 mm (inner diameter) ×100 mm (length), 1.7 μm (particle dimension)) (Waters Corp., MA, USA) was used for separation. The mobile phase was consisted of water (0.1% formic acid, phase A) and acetonitrile (phase B) with a flow rate of 0.3 mL/min and the column temperature was 40°C. The elution gradient was set as follows: 0~1 min，2% B；1~14 min，2%~30% B；14~25 min，30%~100% B；25~28 min，100% B；28~28.1 min，100%~2% B；28.1~30 min，2% B. The flow rate was 0.3 ml/min.

The MS data was collected by a hybrid quadrupole orbitrap mass spectrometer (Q Exactive, Thermo Fisher Scientific, Inc., Waltham, MA, USA) equipped with a HESI-II spray probe. The parameters were set as follows: positive ion source voltage 3.7 kV and negative ion source voltage 3.5 kV, heated capillary temperature 320 °C, sheath gas pressure 30 psi, auxiliary gas pressure 10 psi, desolvation temperature 300 °C. Both the sheath gas and the auxiliary gas were nitrogen. The collision gas was also nitrogen with a pressure of 1.5 mTorr. The data was acquired in “Full scan/dd-MS2” mode. The parameters of the full scan were set as follows: resolution 7000, auto gain control target 1×10^6^, maximum isolation time 50 ms. The dd-MS2 data was collected with the parameters of resolution 17500, auto gain control target 1×10^5^, maximum isolation time 50 ms, loop count of top 10 peaks, isolation window of m/z 2, collision energy 10 V, 30 V, 60 V and intensity threshold 1×10^5^.

The MS data was processed by Progenesis QI 3.0 (Waters Corp., MA, USA) with the steps of raw data introduction, peak extraction and adduct deconvolution. The identification was finally determined by in consideration of retention time error of reference substance, mass error of mother ion, match degree of daughter ions, isotope distribution and peak area after searching the reference substance database (TCM Pro 2.0) and theoretical database constructed by literature and public databases.

**1.2 Animal allocation and exclusion criteria**

Thirty rats were included in the main COPD-like pulmonary injury experiment, with six animals allocated to each experimental group. Biological specimens were allocated to different downstream analyses according to assay requirements. Body-weight measurements, BALF inflammatory-cytokine measurements, colon inflammatory-cytokine measurements, and serum metabolomics were performed using samples from six animals per group. Histopathological and immunofluorescence analyses were performed using three independent biological specimens per group. Lung transcriptomic sequencing, RT-qPCR, and Western blotting were performed using three independent biological specimens per group.

The exclusion criteria were as follows: (1) animals that died during the experimental period; (2) animals that developed unexpected complications unrelated to COPD modeling; (3) samples with confirmed technical failure during processing. In the present study, no animals met these exclusion criteria, and therefore no animals or samples were excluded.

**1.3 Measurement of inflammatory cytokines and enzymes**

The levels of inflammatory cytokines [tumor necrosis factor-α (TNF-α), interleukin-1β (IL-1β), and interleukin-6 (IL-6)] were measured in bronchoalveolar lavage fluid (BALF) and colon samples via enzyme-linked immunosorbent assay (ELISA) kits (Jianglai Biological, Shanghai, China). According to the manufacturer’s instructions, the tissue were lysed and centrifuged at 1000 × g for 20 min to measure the content of inflammatory cytokines. The cells were collected and washed 3 times with PBS and then lysed by ultrasonication at 200 W. Cell debris was removed by centrifugation at 1500 × g for 10 min, and the supernatant were collected for analysis of COX-2 and 5-LOX levels (Jianglai Biological, Shanghai, China).

**1.4 Transcriptomic analyses of lung tissue**

Nine lung tissue samples were selected for high-throughput RNA sequencing. The qualified total RNA samples were digested with DNase I before mRNA enrichment through oligo magnetic beads. The mRNA segments were reverse transcribed into first-strand cDNA through random primers. Then, second-strand cDNA was synthesized. Before adapter ligation, a single "A" base was added, after which single-stranded DNA library enrichment was executed via PCR. After quality control, the raw reads were filtered to obtain clean reads, which were subsequently aligned to the reference genome through HISAT2.

**1.5 Untargeted serum metabolomics analysis**

The untargeted metabolomics analysis was performed by Gene Denovo Biotechnology Co., Ltd. (Guangzhou, China). Quality-control (QC) samples were prepared by pooling equal aliquots of the study samples. QC samples were analyzed before, intermittently during, and after the LC–MS/MS analytical sequence to monitor analytical stability and reproducibility. Biological samples were analyzed in randomized order, and QC samples were inserted into the analytical sequence to monitor system performance throughout data acquisition.

Serum samples were extracted with cold 90% methanol, followed by low-temperature sonication (30 min each time, twice) and centrifugation at 14,000 g for 20 min at 4°C. The supernatants were collected and dried in a vacuum centrifuge. Before LC–MS analysis, the dried extracts were reconstituted in acetonitrile/water (1:1, v/v).

Analysis was performed using an UHPLC (1290 Infinity LC, Agilent Technologies) coupled to a quadrupole time-of-flight (AB Sciex TripleTOF 6600, Shanghai) . For HILIC separation, samples were analyzed using a 2.1 mm × 100 mm ACQUIY UPLC BEH Amide 1.7 µm column (waters, Ireland). In both ESI positive and negative modes, the mobile phase contained A=25 mM ammonium acetate and 25 mM ammonium hydroxide in water and B= acetonitrile. The gradient was 95% B for 0.5 min and was linearly reduced to 65% in 6.5 min, and then was reduced to 40% in 1 min and kept for 1 min, and then increased to 95% in 0.1 min, with a 3 min re-equilibration period employed.The ESI source conditions were set as follows: Ion Source Gas1 (Gas1) as 60, Ion Source Gas2 (Gas2) as 60, curtain gas (CUR) as 30, source temperature: 600℃, IonSpray Voltage Floating (ISVF) ± 5500 V. In MS only acquisition, the instrument was set to acquire over the m/z range 60-1000 Da, and the accumulation time for TOF MS scan was set at 0.20 s/spectra. In auto MS/MS acquisition, the instrument was set to acquire over the m/z range 25-1000 Da, and the accumulation time for product ion scan was set at 0.05 s/spectra. The product ion scan is acquired using information dependent acquisition (IDA) with high sensitivity mode selected. The parameters were set as follows: the collision energy (CE) was fixed at 35 V with ± 15 eV; declustering potential (DP), 60 V (+) and −60 V (−); exclude isotopes within 4 Da, candidate ions to monitor per cycle: 10.

Data acquired in positive- and negative-ionization modes were processed separately. Raw mass-spectrometric data were converted using ProteoWizard MSConvert and subsequently imported into XCMS for peak detection, peak grouping/alignment, retention-time correction, and peak-area extraction. For peak detection, the centWave algorithm was used with an m/z tolerance of 10 ppm, a peak-width range of 10–60 s, and a prefilter setting of (10, 100). For peak grouping, bw = 5, mzwid = 0.025, and minfrac = 0.5 were used. Features with >50% missing values were excluded. Remaining missing values were imputed using the K-nearest-neighbor method, and features with an RSD >50% were removed during quality filtering. Quantitative feature data were subsequently normalized according to the original analytical workflow before downstream statistical analyses.

Metabolite annotation and the associated data processing were performed by Gene Denovo Biotechnology Co., Ltd. (Guangzhou, China). Metabolite features were annotated by comparing accurate precursor-ion m/z values (mass error <10 ppm) and MS/MS spectra with the spectral database/library used in the original analytical workflow.

**Table S1. Identification results of chemical constituents in JGD.**

| **No.** | **Retention time (min)** | **Adducts** | **Experimental m/z** | **Theoretical m/z** | **Mass Error (ppm)** | **Fragments** | **Formula** | **Compound name** |
| --- | --- | --- | --- | --- | --- | --- | --- | --- |
| 1 | 0.77 | [M+H]+ | 175.1187 | 175.1190 | -1.48 | 175.1187, 174.1121, 158.0921, 130.0973, 128.0705, 116.0708, 114.0553 | C6H14N4O2 | Arginine |
| 2 | 0.77 | [M]+ | 104.1075 | 104.1070 | 4.82 | 104.1074, 60.0821, 58.0663, 87.0448 | C5H14NO+ | Choline |
| 3 | 0.77 | [M+H-H2O]+ | 162.0758 | 162.0761 | -1.53 | 162.0757, 144.0652, 126.0550, 100.0249, 98.0607, 97.0291, 96.0451 | C6H13NO5 | D-Glucosamine |
| 4 | 0.81 | [M+H]+ | 148.0601 | 148.0605 | -2.53 | 84.0453, 130.0498, 102.0555, 100.0763 | C5H9NO4 | L-glutamic acid |
| 5 | 0.82 | [M+H-H2O]+ | 487.1649 | 487.1658 | -1.65 | 325.1121, 163.0594, 145.0492, 127.0390, 97.0290, 85.0293, 69.0346 | C18H32O16 | Manninotriose |
| 6 | 0.82 | [M+NH4]+ | 360.1491 | 360.1501 | -2.68 | 325.1116, 163.0597, 145.0492, 127.0389, 97.0290, 85.0293, 69.0347 | C12H22O11 | Lactose |
| 7 | 0.84 | [M+H]+ | 116.0709 | 116.0707 | 2.32 | 70.0663, 116.0708, 71.0695, 72.0818 | C5H9NO2 | L-Proline |
| 8 | 0.87 | [M+FA-H]- | 387.1145 | 387.1144 | 0.11 | 341.1082, 179.0547, 161.0437, 119.0325, 113.0221, 101.0219, 96.9576 | C12H22O11 | Maltose |
| 9 | 0.89 | [M+FA-H]- | 711.2208 | 711.2200 | 1.05 | 665.2162, 485.1507, 383.1217, 341.1083, 323.0980, 179.0546, 161.0436 | C24H42O21 | Nystose |
| 10 | 0.89 | [M+FA-H]- | 549.1674 | 549.1672 | 0.23 | 503.1622, 323.0985, 209.0658, 179.0547, 119.0328, 113.0220, 101.0219 | C18H32O16 | 1-Kestose |
| 11 | 0.90 | [M+H]+ | 144.1017 | 144.1020 | -1.67 | 84.0817, 144.1016, 85.0292, 87.0449 | C7H13NO2 | Stachydrine |
| 12 | 0.91 | [M-H]- | 290.0880 | 290.0881 | -0.34 | 128.0331, 200.0552, 290.0885, 170.0440 | C11H17NO8 | Pyroglutamic acid N-fructoside |
| 13 | 1.21 | [M+FA-H]- | 549.1676 | 549.1672 | 0.63 | 503.1621, 209.0658, 179.0544, 133.0120, 113.0221, 101.0217, 96.9576 | C18H32O16 | Lnulin |
| 14 | 1.23 | [M+H]+ | 136.0616 | 136.0618 | -0.93 | 136.0616, 119.0353, 91.0552, 94.0658 | C5H5N5 | Adenine |
| 15 | 1.23 | [M+FA-H]- | 1197.3801 | 1197.3785 | 1.35 | 1151.3743, 179.0545, 89.0218, 101.0219 | C42H72O36 | 1-Kestoheptaose |
| 16 | 1.25 | [M+H]+ | 123.0555 | 123.0553 | 1.57 | 123.0554, 80.0504, 96.0451, 78.0347 | C6H6N2O | Nicotinamide |
| 17 | 1.41 | [M+FA-H]- | 312.0953 | 312.0949 | 1.26 | 134.0450, 191.0181, 111.0065, 266.0896 | C10H13N5O4 | Adenosine |
| 18 | 1.43 | [M+H]+ | 284.0984 | 284.0990 | -2.05 | 152.0563, 135.0299, 110.0353, 153.0403 | C10H13N5O5 | Guanosine |
| 19 | 1.45 | [M+H]+ | 132.1019 | 132.1020 | -0.16 | 86.0973, 69.0710, 87.1005, 84.0453 | C6H13NO2 | L-isoleucine |
| 20 | 3.10 | [M+H-H2O]+ | 109.0289 | 109.0285 | 3.92 | 109.0287, 107.0859, 98.5126, 87.0048, 81.0344, 77.9997, 67.0554 | C6H6O3 | 5-Hydroxymethylfurfural |
| 21 | 5.24 | [M-H]- | 353.0882 | 353.0878 | 1.09 | 353.0906, 221.1024, 191.0545, 179.0334, 146.9361, 135.0429, 134.0343 | C16H18O9 | Neochlorogenic acid |
| 22 | 5.33 | [M-H]- | 329.0882 | 329.0878 | 1.06 | 123.0429, 329.0879, 167.0332, 149.0224, 81.0320 | C14H18O9 | Vanillic acid glucoside |
| 23 | 7.06 | [M-H]- | 353.0881 | 353.0878 | 0.76 | 191.0547, 353.0873, 85.0269, 93.0319 | C16H18O9 | Chlorogenic acid |
| 24 | 7.41 | [M+H-H2O]+ | 163.0385 | 163.0390 | -2.34 | 163.0386, 135.0439, 117.0338, 89.0394, 145.0282 | C9H8O4 | Caffeic acid |
| 25 | 8.23 | [M+NH4]+ | 420.1852 | 420.1865 | -2.92 | 420.1859, 295.1010, 257.0801, 147.0438, 137.0231, 133.0491, 115.0394 | C18H26O10 | Icariside F2 |
| 26 | 8.87 | [M-H]- | 593.1520 | 593.1511 | 1.38 | 593.1519, 353.0665, 297.0769, 473.1092, 383.0775, 325.0718 | C27H30O15 | Vicenin 2 |
| 27 | 9.09 | [M-H]- | 563.1412 | 563.1406 | 1.04 | 563.1406, 353.0671, 383.0766, 297.0763, 443.0992 | C26H28O14 | Vicenin I |
| 28 | 9.72 | [M+H]+ | 417.1169 | 417.1181 | -2.69 | 255.0646, 199.0753, 417.1175, 137.0231 | C21H20O9 | Daidzin |
| 29 | 9.83 | [M+H]+ | 565.1537 | 565.1552 | -2.56 | 565.1538, 547.1439, 427.1028, 409.0914, 391.0796, 379.0803, 349.0695 | C26H28O14 | Vicenin III |
| 30 | 9.97 | [M+FA-H]- | 433.2081 | 433.2079 | 0.40 | 433.2082, 387.2024, 271.0625, 253.0505, 225.1488, 151.0023, 101.0220 | C19H32O8 | Corchoionoside A |
| 31 | 10.06 | [M-H]- | 677.2307 | 677.2298 | 1.30 | 99.0426, 261.0980, 497.1686, 453.1760 | C29H42O18 | tangshenoside |
| 32 | 10.56 | [M-H]- | 417.1191 | 417.1191 | 0.00 | 255.0661, 119.0479, 135.0065, 417.1198, 91.0164 | C21H22O9 | Liquiritin |
| 33 | 10.56 | [M+FA-H]- | 625.1777 | 625.1774 | 0.48 | 255.0661, 417.1193, 135.0065, 119.0478, 579.1726, 148.0145 | C27H32O14 | Liquiritigenin-7,4'-diglucoside |
| 34 | 10.60 | [M+FA-H]- | 471.2079 | 471.2083 | -0.86 | 471.2087, 425.2036, 271.0611, 263.1505, 161.0436, 151.0021, 113.0220 | C18H34O11 | Hexyl-beta-D-glucopyranosyl-(1->2)-beta-D-glucopyranoside |
| 35 | 10.62 | [M+H-H2O]+ | 389.1215 | 389.1231 | -3.68 | 389.1228, 388.6674, 388.1663, 353.1024, 299.0907, 281.0808, 269.0815 | C20H24O10 | (3r ')-hydroxymarmesin 4'-o-β-d-gluco-pyranoside |
| 36 | 10.80 | [M+H]+ | 433.1112 | 433.1130 | -3.89 | 433.1119, 415.1010, 397.0912, 337.0696, 323.0895, 313.0696, 297.0751 | C21H20O10 | Vitexin |
| 37 | 10.81 | [M-H]- | 549.1612 | 549.1613 | -0.36 | 549.1614, 255.0662, 135.0065, 119.0479, 91.0164 | C26H30O13 | Liquiritin apioside |
| 38 | 10.85 | [M-H]- | 577.1561 | 577.1562 | -0.28 | 577.1572, 457.1142, 383.0776, 353.0669, 297.0767, 295.0608, 115.0741 | C27H30O14 | Isoviolanthin |
| 39 | 10.91 | [M+H]+ | 447.1271 | 447.1286 | -3.36 | 285.0748, 270.0515, 447.1274, 253.0491 | C22H22O10 | Glycitin |
| 40 | 10.95 | [M+H]+ | 433.1115 | 433.1130 | -3.34 | 433.1121, 415.1002, 379.0800, 349.0704, 337.0691, 323.0906, 313.0695 | C21H20O10 | Isovitexin |
| 41 | 11.27 | [M+H]+ | 449.1068 | 449.1079 | -2.31 | 287.0544, 449.1065, 153.0177, 121.0286 | C21H20O11 | Cynaroside |
| 42 | 11.34 | [M+H-H2O]+ | 287.0542 | 287.0551 | -2.80 | 287.0544, 153.0180, 135.0440, 285.0751 | C15H12O7 | cis-Dihydroquercetin |
| 43 | 11.86 | [M-H]- | 431.0984 | 431.0983 | 0.10 | 269.0456, 431.0983, 151.0016, 268.0376 | C21H20O10 | Genistin |
| 44 | 11.96 | [M+FA-H]- | 603.2303 | 603.2294 | 1.59 | 603.2334, 603.0426, 565.1560, 559.2384, 557.2263, 516.0374, 467.1762 | C26H38O13 | Lobetyolinin |
| 45 | 11.97 | [M-H]- | 449.1094 | 449.1089 | 1.12 | 151.0017, 287.0563, 135.0430, 449.1097, 107.0115 | C21H22O11 | Eriodictyol-7-O-glucoside |
| 46 | 12.11 | [M-H]- | 447.0939 | 447.0932 | 1.27 | 447.0925, 255.0294, 284.0327, 285.0392, 227.0339, 160.8400 | C21H20O11 | Trifolin |
| 47 | 12.32 | [M-H]- | 433.1139 | 433.1140 | -0.33 | 271.0613, 433.1138, 151.0017, 119.0479, 107.0117 | C21H22O10 | Prunin |
| 48 | 12.33 | [M+H]+ | 565.1534 | 565.1552 | -3.15 | 271.0593, 565.1540, 153.0177, 121.0281 | C26H28O14 | Apioside |
| 49 | 12.47 | [M+H]+ | 433.1121 | 433.1130 | -1.99 | 271.0594, 433.1113, 153.0180, 273.0733 | C21H20O10 | Sophoricoside |
| 50 | 12.48 | [M-H]- | 431.0985 | 431.0983 | 0.33 | 431.0984, 268.0378, 269.0439, 271.0611 | C21H20O10 | Apigenin-7-glucoside |
| 51 | 13.38 | [M+Na]+ | 447.1273 | 447.1262 | 2.72 | 285.0750, 447.1276, 229.0858, 152.0103 | C20H24O10 | oxymarmesinin 5'-o-β-d-glucopyranoside |
| 52 | 13.38 | [M+H]+ | 551.1744 | 551.1760 | -2.74 | 257.0800, 137.0231, 147.0437, 419.1325 | C26H30O13 | Isoliquiritin apioside |
| 53 | 13.40 | [M+FA-H]- | 441.1766 | 441.1766 | -0.13 | 441.1767, 414.8363, 305.1244, 270.1443, 215.1067, 189.3457, 185.0950 | C20H28O8 | Lobetyolin |
| 54 | 13.49 | [M+H-H2O]+ | 445.1117 | 445.1130 | -2.69 | 283.0594, 225.0543, 253.0489, 285.0748 | C22H22O11 | 7alpha-L-Rhamnosyl-6-methoxyluteolin |
| 55 | 13.58 | [M+H]+ | 563.1742 | 563.1760 | -3.01 | 269.0801, 563.1750, 254.0565, 213.0906 | C27H30O13 | Glycyroside |
| 56 | 13.71 | [M+H]+ | 461.1428 | 461.1443 | -3.03 | 257.0802, 137.0231, 147.0438, 81.0348 | C23H24O10 | 6''-O-Acetylliquiritin |
| 57 | 13.91 | [M+Na]+ | 269.0794 | 269.0785 | 3.95 | 269.0801, 254.0564, 237.0541, 213.0904 | C14H14O4 | (+/-)-Marmesin |
| 58 | 13.94 | [M+H]+ | 431.1323 | 431.1337 | -3.07 | 269.0801, 431.1331, 254.0565, 213.0906 | C22H22O9 | Ononin |
| 59 | 14.03 | [M+H]+ | 419.1325 | 419.1337 | -2.80 | 257.0801, 137.0231, 147.0437, 419.1327 | C21H22O9 | Isoliquiritin |
| 60 | 14.19 | [M+H]+ | 255.0645 | 255.0652 | -2.56 | 255.0645, 137.0231, 199.0750, 147.0437 | C15H10O4 | Daidzein |
| 61 | 14.27 | [M+H-H2O]+ | 1249.5817 | 1249.5848 | -2.43 | 1249.5796, 1103.5254, 927.4943, 733.4506, 571.3998, 439.3568, 421.3442 | C59H96O30 | Platycoside G2 |
| 62 | 14.27 | [M+H]+ | 257.0799 | 257.0809 | -3.69 | 257.0800, 137.0231, 147.0437, 119.0494 | C15H12O4 | Liquiritigenin |
| 63 | 14.52 | [M+H]+ | 301.0697 | 301.0707 | -3.06 | 301.0699, 137.0231, 213.0536, 286.0464 | C16H12O6 | 7,2',4'-trihydroxy-5-methoxy-3-phenylcoumarin |
| 64 | 14.61 | [M+FA-H]- | 1299.5878 | 1299.5862 | 1.20 | 1253.5825, 843.4406, 519.3332, 471.3116 | C58H94O29 | Deapio-Platycodin-D3 |
| 65 | 14.82 | [M+H]+ | 449.1431 | 449.1443 | -2.51 | 449.1430, 287.0908, 177.0544, 151.0387, 145.0281, 137.0232, 121.0287 | C22H24O10 | 4''-hydroxyimperatorin 4''-o-β-d-glucopyra-noside |
| 66 | 14.84 | [M+FA-H]- | 1431.6304 | 1431.6285 | 1.32 | 1385.6244, 469.1564, 843.4401, 131.0327 | C63H102O33 | Platycodin D3 |
| 67 | 15.26 | [M+H]+ | 1239.5977 | 1239.6005 | -2.25 | 1107.5594, 975.5120, 961.4930, 945.5075, 829.4597, 813.4592, 783.4492 | C58H94O28 | Platycoside H |
| 68 | 15.27 | [M+FA-H]- | 1415.6361 | 1415.6336 | 1.81 | 1369.6300, 469.1564, 827.4454, 131.0329 | C63H102O32 | Platycoside G3 |
| 69 | 15.30 | [M+H]+ | 285.0748 | 285.0758 | -3.18 | 285.0750, 270.0515, 137.0231, 225.0542 | C16H12O5 | Calycosin |
| 70 | 15.38 | [M-H]- | 841.4229 | 841.4227 | 0.18 | 841.4235, 840.4114, 839.4076, 351.0573, 193.0339, 113.0220, 99.0062 | C42H66O17 | Ginseng |
| 71 | 15.80 | [M+H]+ | 845.4494 | 845.4530 | -4.14 | 845.4512, 770.9847, 683.3939, 521.3475, 503.3336, 485.3268, 467.3168 | C42H68O17 | Platycoside L |
| 72 | 16.05 | [M-H]- | 1253.5823 | 1253.5807 | 1.18 | 1253.5822, 843.4410, 519.3327, 113.0221 | C58H94O29 | Platycoside A |
| 73 | 16.15 | [M+H]+ | 961.4979 | 961.5003 | -2.48 | 961.5005, 829.4503, 815.4386, 799.4455, 683.3981, 667.4019, 653.3885 | C47H76O20 | Deapi-platycoside F |
| 74 | 16.17 | [M+FA-H]- | 1137.5356 | 1137.5334 | 1.94 | 1091.5303, 681.3867, 337.1146, 131.0327 | C52H84O24 | Deapio-Platycodin-D |
| 75 | 16.22 | [M-H]- | 1385.6227 | 1385.6230 | -0.22 | 1385.6249, 843.4404, 469.1564, 113.0220 | C63H102O33 | Platycodin D2 |
| 76 | 16.27 | [M-H]- | 725.2098 | 725.2087 | 1.55 | 725.2109, 531.1509, 255.0662, 193.0493, 175.0384, 160.0147, 148.0146 | C36H38O16 | Licorice glycoside a |
| 77 | 16.34 | [M-H]- | 1369.6269 | 1369.6281 | -0.91 | 1369.6299, 469.1566, 827.4451, 113.0221 | C63H102O32 | Polygalacin D2 |
| 78 | 16.34 | [M-H]- | 1223.5697 | 1223.5702 | -0.46 | 1223.5714, 469.1563, 681.3865, 131.0327 | C57H92O28 | Platycodin D |
| 79 | 16.35 | [M+H]+ | 845.4502 | 845.4530 | -3.27 | 845.4584, 683.3978, 521.3471, 503.3365, 485.3257, 455.3140, 421.3056 | C42H68O17 | Platycoside K |
| 80 | 16.35 | [M+H]+ | 273.0748 | 273.0758 | -3.42 | 273.0751, 153.0179, 137.0231, 147.0438, 163.0386, 121.0286 | C15H12O5 | Naringenin |
| 81 | 16.51 | [M-H]- | 1207.5760 | 1207.5753 | 0.58 | 1207.5770, 469.1564, 665.3914, 131.0327, 113.0220 | C57H92O27 | Polygalacin D |
| 82 | 16.51 | [M-H]- | 1237.5495 | 1237.5494 | 0.00 | 1237.5513, 1027.4766, 1207.5425, 485.2908 | C57H90O29 | Platyconic acid A |
| 83 | 16.51 | [M+H]+ | 271.0595 | 271.0601 | -2.25 | 271.0596, 153.0180, 121.0287, 215.0701 | C15H10O5 | Genistein |
| 84 | 16.67 | [M+H]+ | 271.0955 | 271.0965 | -3.77 | 121.0286, 271.0959, 271.0598, 229.0854, 107.0496, 177.0543 | C16H14O4 | Echinatin |
| 85 | 16.83 | [M+H-H2O]+ | 1117.5400 | 1117.5426 | -2.20 | 1117.5444, 971.4822, 809.4292, 615.3887, 471.3467, 453.3354, 421.3458 | C54H86O25 | Platycoside C |
| 86 | 16.85 | [M-H]- | 1265.5814 | 1265.5807 | 0.49 | 1265.5823, 681.3868, 469.1563, 131.0327 | C59H94O29 | 3''O-Acetylplatycodin D |
| 87 | 16.85 | [M+H]+ | 683.3983 | 683.4002 | -2.60 | 683.4021, 521.3458, 503.3388, 485.3246, 455.3153, 449.3057, 213.1633 | C36H58O12 | 3-o-β-d-glucopyranosyl platycodigenin |
| 88 | 16.97 | [M+H-H2O]+ | 517.3149 | 517.3160 | -2.09 | 517.3151, 469.2937, 203.1427, 187.1478, 145.1009, 133.1011, 131.0855 | C30H46O8 | Platycogenic acid B |
| 89 | 17.01 | [M-H]- | 1249.5859 | 1249.5858 | 0.02 | 1249.5872, 665.3915, 469.1564, 113.0220, 131.0328 | C59H94O28 | 3''-O-Acetylpolygalacin D |
| 90 | 17.08 | [M+H]+ | 985.4604 | 985.4639 | -3.59 | 985.4624, 809.4310, 615.3876, 453.3355, 121.1013, 107.0860, 95.0863 | C48H72O21 | licoricesaponine a3 |
| 91 | 17.08 | [M+H]+ | 823.4083 | 823.4111 | -3.34 | 453.3356, 647.3778, 95.0863, 107.0860, 121.1014 | C42H62O16 | Glyyunnanprosapogenin D |
| 92 | 17.26 | [M-H]- | 955.4547 | 955.4544 | 0.34 | 955.4587, 659.3453, 85.0268, 71.0112, 501.3228, 99.0063 | C47H72O20 | Platycoside M2 |
| 93 | 17.29 | [M-H]- | 1011.4821 | 1011.4806 | 1.43 | 1011.4827, 967.4565, 71.0112, 113.0221, 85.0269, 951.4609 | C50H76O21 | Licoricesaponine d3 |
| 94 | 17.42 | [M+H]+ | 301.0696 | 301.0707 | -3.52 | 301.0706, 286.0462, 285.0374, 207.0285 | C16H12O6 | Diosmetin |
| 95 | 17.51 | [M+H]+ | 839.4029 | 839.4060 | -3.64 | 839.4053, 663.3737, 469.3304, 451.3216, 173.1317, 141.0180, 121.1012 | C42H62O17 | licoricesaponin g2 |
| 96 | 17.67 | [M+H-H2O]+ | 337.1058 | 337.1071 | -3.64 | 337.1061, 279.0643, 283.0593, 293.1281 | C20H18O6 | Gancaonin C |
| 97 | 17.68 | [M-H]- | 255.0660 | 255.0662 | -1.19 | 119.0479, 255.0662, 135.0065, 91.0164, 153.0174 | C15H12O4 | Isoliquiritigenin |
| 98 | 18.05 | [M+H]+ | 269.0798 | 269.0809 | -3.82 | 269.0803, 254.0564, 213.0906, 253.0487 | C16H12O4 | Formononetin |
| 99 | 18.09 | [M+H]+ | 373.1269 | 373.1282 | -3.31 | 373.1270, 371.1479, 357.1689, 343.0807, 315.0848, 153.0543, 69.0711 | C20H20O7 | Isosinensetin |
| 100 | 18.25 | [M+H]+ | 353.1008 | 353.1020 | -3.43 | 353.1010, 311.0545, 299.0543, 153.0181, 351.2130 | C20H16O6 | Semilicoisoflavone b |
| 101 | 18.50 | [M-H]- | 821.3975 | 821.3965 | 1.19 | 821.3982, 351.0573, 193.0340, 113.0220, 85.0270, 75.0062, 72.9905 | C42H62O16 | Glycyrrhizic acid |
| 102 | 18.67 | [M-H]- | 819.3833 | 819.3808 | 2.96 | 351.0571, 113.0220, 193.0340, 819.3820 | C42H60O16 | Licoricesaponin e2 |
| 103 | 18.71 | [M+FA-H]- | 369.1342 | 369.1343 | -0.35 | 369.1359, 368.1219, 351.1252, 229.0863, 174.0306, 139.0379, 124.0143 | C20H20O4 | (2S)-7-hydroxy-2-(4-hydroxyphenyl)-8-(3-methylbut-2-enyl)chroman-4-one |
| 104 | 18.79 | [M+H]+ | 1013.4916 | 1013.4952 | -3.54 | 867.4356, 497.3640, 437.3404, 419.3302, 353.0710, 189.1632, 173.1321 | C50H76O21 | licoricesaponin d3 |
| 105 | 18.94 | [M-H]- | 821.3978 | 821.3965 | 1.60 | 821.3983, 351.0571, 113.0220, 193.0341, 85.0269, 71.0112 | C42H62O16 | Uralsaponin B |
| 106 | 18.99 | [M+H]+ | 943.5229 | 943.5261 | -3.42 | 943.5257, 797.4649, 635.4135, 617.4041, 605.4384, 599.3950, 441.3730 | C48H78O18 | Soyasaponin Bb |
| 107 | 19.10 | [M+H]+ | 323.1267 | 323.1278 | -3.47 | 323.1267, 267.0641, 268.0697, 167.0336 | C20H18O4 | Glabrene |
| 108 | 19.19 | [M+H]+ | 355.1524 | 355.1540 | -4.45 | 355.1528, 353.1380, 313.1425, 299.0902, 193.0492, 189.0907, 178.0257 | C21H22O5 | Licochalcone D |
| 109 | 19.28 | [M+H-H2O]+ | 339.1217 | 339.1227 | -2.80 | 339.1218, 283.0594, 271.0595, 255.0636, 205.0857, 203.1063, 149.0229 | C20H20O6 | Sigmoidin b |
| 110 | 19.29 | [M-H]- | 777.4080 | 777.4066 | 1.69 | 777.4097, 59.0112, 71.0112, 99.0063, 627.3564 | C41H62O14 | Apioglycyrrhizin |
| 111 | 19.37 | [M+H]+ | 825.4230 | 825.4268 | -4.49 | 825.4241, 455.3510, 453.3353, 437.3401, 353.0708, 203.1787, 189.1634 | C42H64O16 | Licorice-saponin j2 |
| 112 | 19.41 | [M+H]+ | 355.1163 | 355.1177 | -3.63 | 355.1143, 354.1043, 312.0576, 201.0907, 189.0905, 179.0336, 153.0182 | C20H18O6 | Licoisoflavone A |
| 113 | 19.43 | [M+H]+ | 343.1167 | 343.1177 | -2.73 | 343.1169, 313.0698, 299.0909, 285.0749, 282.0876, 205.0856, 121.0286 | C19H18O6 | Scutellarein tetramethyl ether |
| 114 | 19.46 | [M+H]+ | 403.1374 | 403.1388 | -3.44 | 403.1382, 373.0909, 388.1140, 211.0236 | C21H22O8 | Nobiletin |
| 115 | 19.48 | [M+H]+ | 369.1319 | 369.1333 | -3.77 | 369.1325, 313.0701, 285.0749, 351.1218 | C21H20O6 | Glycyrrhisoflavanone |
| 116 | 19.48 | [M+H]+ | 357.1321 | 357.1333 | -3.37 | 357.1319, 355.1164, 301.1060, 301.0699, 191.1063, 175.0387, 165.0544 | C20H20O6 | Sigmoidin-B |
| 117 | 19.55 | [M-H]- | 805.4033 | 805.4015 | 2.10 | 805.4042, 351.0571, 113.0220, 193.0336 | C42H62O15 | Licoricesaponine c2 |
| 118 | 19.63 | [M+H]+ | 341.1373 | 341.1384 | -2.97 | 341.1368, 340.1254, 285.0715, 284.0627, 283.0593, 189.0905, 183.0285 | C20H20O5 | 2,4,2',4'-tetrahydroxy-3'-prenylchalcone |
| 119 | 19.68 | [M+H]+ | 807.4135 | 807.4162 | -3.24 | 437.3407, 631.3820, 95.0862, 107.0860 | C42H62O15 | Licoricesaponin C2 |
| 120 | 19.70 | [M+H]+ | 353.1009 | 353.1020 | -2.93 | 353.1008, 153.0179, 299.0542, 299.0905 | C20H16O6 | Licoisoflavone B |
| 121 | 19.72 | [M-H]- | 321.1133 | 321.1132 | 0.23 | 321.1133, 175.0746, 107.0478, 306.0899 | C20H18O4 | Licoflavone a |
| 122 | 19.77 | [M+H]+ | 315.0852 | 315.0864 | -3.49 | 315.0854, 300.0618, 257.0802, 299.0544 | C17H14O6 | Velutin |
| 123 | 19.77 | [M-H]- | 807.4181 | 807.4172 | 1.03 | 807.4193, 351.0573, 193.0341, 113.0220, 85.0269, 75.0061, 72.9905 | C42H64O15 | Licoricesaponin b2 |
| 124 | 19.96 | [M-H]- | 339.1238 | 339.1237 | -0.07 | 339.1239, 324.1006, 165.0176, 281.0460, 269.0456 | C20H20O5 | Licocoumarone |
| 125 | 19.99 | [M+H]+ | 441.1897 | 441.1908 | -2.39 | 441.1897, 439.3559, 423.1785, 385.1277, 367.1159, 365.1007, 313.0694 | C25H28O7 | Kanzonol t |
| 126 | 20.01 | [M+H]+ | 355.1164 | 355.1177 | -3.35 | 355.1168, 337.1065, 299.0546, 229.0854, 201.0907, 189.0910, 179.0336 | C20H18O6 | 6-Prenylluteolin |
| 127 | 20.09 | [M-H]- | 235.0968 | 235.0975 | -3.13 | 235.0970, 177.0177, 165.0175, 152.0094, 205.0487, 220.0730 | C13H16O4 | 3-Butyl-4-methylphthalic acid |
| 128 | 20.10 | [M-H]- | 353.1030 | 353.1030 | -0.05 | 125.0221, 353.1031, 57.0320, 227.0708 | C20H18O6 | Glyasperin f |
| 129 | 20.16 | [M+H]+ | 341.1375 | 341.1384 | -2.56 | 341.1375, 341.1307, 340.1258, 285.0751, 284.0656, 267.0645, 173.0594 | C20H20O5 | Glepidotin b |
| 130 | 20.30 | [M+H]+ | 383.1478 | 383.1490 | -3.00 | 327.0856, 383.1486, 299.0914, 178.0623 | C22H22O6 | Glycyrin |
| 131 | 20.34 | [M+H]+ | 355.1527 | 355.1540 | -3.62 | 355.1534, 299.0574, 215.1064, 191.1063, 153.0543, 149.0595, 135.0440 | C21H22O5 | Dehydroglyasperin c |
| 132 | 20.35 | [M-H]- | 337.1085 | 337.1081 | 1.16 | 337.1081, 151.0016, 187.1113, 281.0453 | C20H18O5 | Yinyanghuo D |
| 133 | 20.36 | [M+H]+ | 357.1682 | 357.1697 | -3.98 | 357.1652, 356.1571, 301.1062, 191.1064, 165.0543, 153.0543, 149.0596 | C21H24O5 | Glyasperin C |
| 134 | 20.38 | [M+H]+ | 647.3775 | 647.3790 | -2.33 | 453.3357, 647.3809, 95.0863, 107.0860 | C36H54O10 | Glycyrrhetic acid 3-O-β-D-glucuronide |
| 135 | 20.43 | [M+H-H2O]+ | 367.1165 | 367.1177 | -2.80 | 367.1166, 311.0540, 296.0309, 281.0435 | C21H20O7 | Gancaonin P-3'-methylether |
| 136 | 20.70 | [M-H]- | 335.0928 | 335.0924 | 0.76 | 335.0929, 291.1024, 135.0065, 320.0674 | C20H16O5 | Glabrone |
| 137 | 20.71 | [M+H-H2O]+ | 337.1059 | 337.1071 | -3.20 | 337.1062, 283.0594, 295.0598, 137.0232 | C20H18O6 | Isolicoflavonol |
| 138 | 20.71 | [M+H]+ | 325.1425 | 325.1435 | -2.76 | 123.0442, 325.1425, 189.0907, 149.0595, 137.0232, 147.0806 | C20H20O4 | Corylifolinin |
| 139 | 20.74 | [M-H]- | 323.1290 | 323.1288 | 0.38 | 323.1290, 135.0430, 201.0909, 109.0271 | C20H20O4 | Glabridin |
| 140 | 20.87 | [M-H]- | 485.3278 | 485.3272 | 1.21 | 485.3277, 483.3125, 441.3401, 453.3003 | C30H46O5 | 24-hydroxyglycyrrhetic acid |
| 141 | 21.00 | [M-H]- | 423.1820 | 423.1813 | 1.62 | 423.1818, 395.1867, 193.0856, 124.0144 | C25H28O6 | Gancaonin E |
| 142 | 21.59 | [M+H]+ | 369.1327 | 369.1333 | -1.51 | 369.1317, 368.1598, 367.1540, 353.1379, 313.0699, 309.0752, 189.0902 | C21H20O6 | Glyasperin M |
| 143 | 21.59 | [M+H]+ | 371.1842 | 371.1853 | -2.91 | 371.1841, 369.1326, 315.1216, 313.0696, 303.1219, 235.1323, 193.0858 | C22H26O5 | Glyasperin d |
| 144 | 21.81 | [M+H]+ | 421.1637 | 421.1646 | -1.96 | 421.1633, 365.1011, 189.0908, 165.0179, 323.0540 | C25H24O6 | Gancaonin h |
| 145 | 21.81 | [M+H]+ | 423.1796 | 423.1803 | -1.56 | 423.1781, 422.1657, 367.1172, 349.1051, 313.0699, 295.0583, 221.1168 | C25H26O6 | Isoangustone A |
| 146 | 21.92 | [M+H]+ | 485.3253 | 485.3262 | -1.78 | 485.3274, 467.3158, 233.1532, 187.1480, 173.1323, 145.1011, 119.0858 | C30H44O5 | Liquoric acid |
| 147 | 22.09 | [M-H]- | 423.1820 | 423.1813 | 1.52 | 193.0857, 423.1815, 229.0864, 149.0950 | C25H28O6 | 3'-(γ,γ-dimethylallyl)-kievitone |
| 148 | 22.12 | [M-H]- | 421.1662 | 421.1656 | 1.28 | 193.0856, 421.1662, 229.0863, 149.0953 | C25H26O6 | Glyasperin a |
| 149 | 22.25 | [M+H]+ | 297.1478 | 297.1486 | -2.59 | 297.1479, 254.0930, 251.1425, 282.1245 | C19H20O3 | Cryptotanshinone |
| 150 | 22.64 | [M+H]+ | 353.1375 | 353.1384 | -2.37 | 297.0755, 353.1376, 267.0644, 299.0545 | C21H20O5 | Gancaonin a |
| 151 | 23.04 | [M+H-H2O]+ | 407.1846 | 407.1853 | -1.72 | 295.0596, 351.1220, 407.1844, 177.0184 | C25H28O6 | Glisoflavanone |
| 152 | 23.17 | [M+H]+ | 471.3462 | 471.3469 | -1.57 | 471.3465, 95.0860, 121.1015, 189.1632 | C30H46O4 | 18alpha-glycyrrhetinic acid |
| 153 | 23.35 | [M+H]+ | 407.1846 | 407.1853 | -1.62 | 407.1845, 351.1217, 205.0856, 189.0907, 187.1115, 183.0287, 165.0180 | C25H26O5 | E)-1-[2,4-dihydroxy-3-(3-methylbut-2-enyl)phenyl]-3-(8-hydroxy-2,2-dimethylchromen-6-yl)prop-2-en-1-one |
| 154 | 23.45 | [M+H]+ | 295.1323 | 295.1329 | -2.05 | 295.1324, 277.1214, 249.1268, 252.0772, 262.0986, 266.0937 | C19H18O3 | Tanshinone IIA |
| 155 | 23.68 | [M+H]+ | 421.1636 | 421.1646 | -2.35 | 365.1013, 165.0179, 421.1635, 183.0285 | C25H24O6 | Morusin |
| 156 | 23.79 | [M+H]+ | 469.3306 | 469.3313 | -1.40 | 469.3307, 95.0862, 149.0961, 121.1013 | C30H44O4 | Glabrolide |

**Table S2. Identification results of the constituents derived from JGD adsorbed into the serum.**

| **No.** | **Parent compound** | **Transformation** | **Retention time (min)** | **Adducts** | **Experimental m/z** | **Theoretical m/z** | **Mass Error (ppm)** |
| --- | --- | --- | --- | --- | --- | --- | --- |
| 1 | (2S)-7-hydroxy-2-(4-hydroxyphenyl)-8-(3-methylbut-2-enyl)chroman-4-one |  | 18.70 | M+FA-H | 369.1345 | 369.1344 | 0.47 |
| 2 | (3r ')-hydroxymarmesin 4'-o-β-d-gluco-pyranoside |  | 10.31 | M+Na | 447.1281 | 447.1262 | 4.35 |
| 3 | 24-hydroxyglycyrrhetic acid |  | 20.87 | M-H | 485.3279 | 485.3272 | 1.29 |
| 4 | 3-O-beta-D-Glucopyranosylplatycodigenin |  | 16.34 | M+H | 683.3987 | 683.4001 | -2.04 |
| 5 | 4''-hydroxyimperatorin 4''-o-β-d-glucopyra-noside |  | 14.81 | M-H | 447.1302 | 447.1297 | 1.20 |
| 6 | 4''-hydroxyimperatorin 4''-o-β-d-glucopyra-noside | -H2 | 15.92 | M-H | 445.1146 | 445.1140 | 1.24 |
| 7 | 7,2',4'-trihydroxy-5-methoxy-3-phenylcoumarin |  | 14.75 | M-H | 299.0563 | 299.0561 | 0.48 |
| 8 | 7alpha-L-Rhamnosyl-6-methoxyluteolin |  | 12.91 | M+H | 463.1227 | 463.1235 | -1.68 |
| 9 | Apigenin-7-glucoside |  | 12.89 | M+FA-H | 477.1041 | 477.1039 | 0.62 |
| 10 | Apioglycyrrhizin | +C6H8O6 | 16.55 | M+FA-H | 999.4464 | 999.4443 | 2.11 |
| 11 | Apioglycyrrhizin | +O | 17.15 | M+FA-H | 839.4089 | 839.4071 | 2.23 |
| 12 | Apioglycyrrhizin |  | 19.29 | M-H | 777.4085 | 777.4067 | 2.38 |
| 13 | cis-Dihydroquercetin |  | 11.24 | M+H | 305.0648 | 305.0656 | -2.58 |
| 14 | Corchoionoside A |  | 10.55 | M-H | 387.2027 | 387.2024 | 0.66 |
| 15 | Corylifolinin |  | 20.72 | M-H | 323.1292 | 323.1289 | 0.86 |
| 16 | Cynaroside |  | 10.92 | M+H | 449.1071 | 449.1078 | -1.57 |
| 17 | Daidzin |  | 9.71 | M+H | 417.1172 | 417.1180 | -1.83 |
| 18 | Daidzin | +O | 8.10 | M+H | 433.1126 | 433.1129 | -0.82 |
| 19 | Deapio-Platycodin-D |  | 16.34 | M+H | 1093.5397 | 1093.5425 | -2.59 |
| 20 | Deapio-Platycodin-D | +H2 | 14.61 | M+Na | 1117.5399 | 1117.5401 | -0.17 |
| 21 | Deapio-Platycodin-D3 |  | 14.83 | M+H | 1255.5928 | 1255.5954 | -2.06 |
| 22 | Deapi-platycoside F |  | 16.34 | M+H | 961.4983 | 961.5003 | -2.06 |
| 23 | E)-1-[2,4-dihydroxy-3-(3-methylbut-2-enyl)phenyl]-3-(8-hydroxy-2,2-dimethylchromen-6-yl)prop-2-en-1-one |  | 23.03 | M+H | 407.1843 | 407.1853 | -2.44 |
| 24 | Echinatin |  | 15.90 | M+H | 271.0957 | 271.0965 | -2.76 |
| 25 | Eriodictyol-7-O-glucoside |  | 11.23 | M-H | 449.1093 | 449.1089 | 0.77 |
| 26 | Gancaonin C |  | 17.45 | M+H | 355.1163 | 355.1176 | -3.78 |
| 27 | Gancaonin E |  | 21.80 | M+H | 425.1947 | 425.1959 | -2.76 |
| 28 | Genistin |  | 11.54 | M-H | 431.0985 | 431.0984 | 0.33 |
| 29 | Genistin | -COO | 13.96 | M+FA-H | 433.1143 | 433.1140 | 0.60 |
| 30 | Genistin | +SO3 | 9.39 | M-H | 511.0553 | 511.0552 | 0.29 |
| 31 | Glabrolide |  | 23.81 | M+H | 469.3305 | 469.3312 | -1.57 |
| 32 | Glyasperin C |  | 19.62 | M-H | 355.1554 | 355.1551 | 0.84 |
| 33 | Glyasperin f |  | 20.11 | M-H | 353.1035 | 353.1031 | 1.24 |
| 34 | Glyasperin M |  | 20.60 | M+H | 369.1325 | 369.1333 | -2.13 |
| 35 | Glycitin |  | 10.29 | M+FA-H | 491.1199 | 491.1195 | 0.75 |
| 36 | Glycyrin |  | 20.29 | M+H | 383.1477 | 383.1489 | -3.22 |
| 37 | Glycyroside | +O | 10.84 | M+H | 579.1700 | 579.1708 | -1.39 |
| 38 | Glycyroside |  | 13.58 | M+H | 563.1751 | 563.1759 | -1.52 |
| 39 | Glycyrrhetic acid 3-O-β-D-glucuronide | +C6H8O6 | 16.90 | M-H | 821.3989 | 821.3965 | 2.90 |
| 40 | Glycyrrhetic acid 3-O-β-D-glucuronide | +O | 18.08 | M+H | 663.3713 | 663.3739 | -3.93 |
| 41 | Glycyrrhetic acid 3-O-β-D-glucuronide |  | 20.40 | M+H | 647.3771 | 647.3790 | -2.83 |
| 42 | Glycyrrhisoflavanone |  | 19.35 | M+H | 369.1320 | 369.1333 | -3.46 |
| 43 | Glyyunnanprosapogenin D |  | 16.93 | M+H | 823.4077 | 823.4111 | -4.07 |
| 44 | Hexyl-beta-D-glucopyranosyl-(1->2)-beta-D-glucopyranoside |  | 10.98 | M+FA-H | 471.2082 | 471.2083 | -0.18 |
| 45 | Icariside F2 |  | 8.16 | M+FA-H | 447.1512 | 447.1508 | 0.87 |
| 46 | Isoliquiritigenin |  | 17.68 | M-H | 255.0663 | 255.0663 | -0.13 |
| 47 | Isoliquiritin | +C6H8O6 | 10.80 | M+FA-H | 639.1573 | 639.1567 | 0.98 |
| 48 | Isoliquiritin |  | 14.04 | M+H | 419.1327 | 419.1337 | -2.22 |
| 49 | Isoliquiritin apioside |  | 13.68 | M+H | 551.1750 | 551.1759 | -1.68 |
| 50 | Isoliquiritin apioside | +SO3 | 9.69 | M-H | 629.1190 | 629.1182 | 1.29 |
| 51 | Isoliquiritin apioside | +C6H8O6 | 8.42 | M-H | 725.1949 | 725.1935 | 2.05 |
| 52 | Isoliquiritin apioside | +O-H2 | 10.92 | M+FA-H | 609.1471 | 609.1461 | 1.66 |
| 53 | Isoviolanthin |  | 10.86 | M-H | 577.1568 | 577.1563 | 0.84 |
| 54 | Isovitexin |  | 10.59 | M-H | 431.0983 | 431.0984 | -0.10 |
| 55 | Licoisoflavone B | +C6H8O6 | 17.29 | M-H | 527.1199 | 527.1195 | 0.84 |
| 56 | Licoisoflavone B |  | 19.23 | M+H | 353.1005 | 353.1020 | -4.15 |
| 57 | Licoricesaponin a3 | +H2 | 16.82 | M-H | 985.4672 | 985.4650 | 2.20 |
| 58 | Licoricesaponin a3 |  | 17.09 | M-H | 983.4513 | 983.4493 | 1.97 |
| 59 | Licoricesaponin b2 |  | 19.77 | M-H | 807.4193 | 807.4172 | 2.50 |
| 60 | Licoricesaponin b2 | +O | 16.27 | M+H | 825.4246 | 825.4267 | -2.55 |
| 61 | Licoricesaponin b2 | +CO | 18.03 | M+FA-H | 881.4193 | 881.4176 | 1.90 |
| 62 | licoricesaponin d3 |  | 18.17 | M-H | 1011.4829 | 1011.4806 | 2.24 |
| 63 | licoricesaponin d3 | +O-H2 | 17.17 | M-H | 1025.4626 | 1025.4599 | 2.60 |
| 64 | licoricesaponin d3 | +H2 | 17.73 | M+FA-H | 1059.5043 | 1059.5018 | 2.39 |
| 65 | Licoricesaponin e2 |  | 18.01 | M-H | 819.3829 | 819.3809 | 2.44 |
| 66 | licoricesaponin g2 | +O | 15.05 | M+H | 855.3991 | 855.4009 | -2.11 |
| 67 | licoricesaponin g2 |  | 17.25 | M+H | 839.4034 | 839.4060 | -3.03 |
| 68 | licoricesaponin j2 | -H2O | 20.39 | M-H | 805.4034 | 805.4016 | 2.29 |
| 69 | licoricesaponin j2 |  | 17.76 | M-H | 823.4140 | 823.4122 | 2.24 |
| 70 | Licorice-saponin j2 |  | 19.48 | M+Na | 847.4063 | 847.4087 | -2.81 |
| 71 | licoricesaponine a3 |  | 17.47 | M+H | 985.4627 | 985.4639 | -1.25 |
| 72 | Licoricesaponine c2 | +CO | 17.40 | M+FA-H | 879.4040 | 879.4020 | 2.33 |
| 73 | Licoricesaponine c2 |  | 19.62 | M-H | 805.4037 | 805.4016 | 2.55 |
| 74 | Licoricesaponine c2 | +H2 | 18.94 | M-H | 807.4196 | 807.4172 | 2.94 |
| 75 | Licoricesaponine c2 | +H2O | 16.28 | M-H | 823.4144 | 823.4122 | 2.75 |
| 76 | Licoricesaponine d3 |  | 17.14 | M+FA-H | 1057.4891 | 1057.4861 | 2.81 |
| 77 | Liquiritigenin |  | 14.26 | M-H | 255.0660 | 255.0663 | -0.98 |
| 78 | Liquiritigenin-7,4'-diglucoside |  | 9.75 | M-H | 579.1725 | 579.1719 | 0.95 |
| 79 | Liquiritin |  | 10.77 | M+H | 419.1327 | 419.1337 | -2.20 |
| 80 | Liquiritin | +CO | 8.99 | M+H | 447.1280 | 447.1286 | -1.37 |
| 81 | Liquiritin | -H2 | 8.45 | M-H | 415.1038 | 415.1035 | 0.90 |
| 82 | Liquiritin apioside |  | 10.77 | M+Na | 573.1568 | 573.1579 | -1.79 |
| 83 | Liquoric acid |  | 21.58 | M+H | 485.3241 | 485.3262 | -4.28 |
| 84 | Lobetyolin |  | 13.38 | M+FA-H | 441.1770 | 441.1766 | 0.75 |
| 85 | Lobetyolinin |  | 11.97 | M+FA-H | 603.2306 | 603.2294 | 1.84 |
| 86 | oxymarmesinin 5'-o-β-d-glucopyranoside |  | 14.28 | M+Na | 447.1276 | 447.1262 | 3.17 |
| 87 | Platycodin D |  | 16.33 | M-H | 1223.5711 | 1223.5702 | 0.72 |
| 88 | Platycodin D2 |  | 16.24 | M-H | 1385.6237 | 1385.6231 | 0.44 |
| 89 | Platycodin D3 |  | 14.84 | M+FA-H | 1431.6312 | 1431.6285 | 1.84 |
| 90 | Platyconic acid A |  | 16.51 | M-H | 1237.5510 | 1237.5495 | 1.16 |
| 91 | Platycoside A |  | 16.04 | M-H | 1253.5831 | 1253.5808 | 1.83 |
| 92 | Platycoside G2 |  | 15.06 | M-H | 1283.5932 | 1283.5914 | 1.41 |
| 93 | Platycoside G3 |  | 15.27 | M+FA-H | 1415.6364 | 1415.6336 | 1.95 |
| 94 | Platycoside H |  | 16.20 | M+FA-H | 1283.5936 | 1283.5914 | 1.75 |
| 95 | Platycoside L |  | 14.83 | M+H | 845.4505 | 845.4529 | -2.88 |
| 96 | Platycoside M2 |  | 16.92 | M+FA-H | 1001.4623 | 1001.4599 | 2.42 |
| 97 | Polygalacin D |  | 16.51 | M-H | 1207.5763 | 1207.5753 | 0.77 |
| 98 | Prunin |  | 12.98 | M-H | 433.1141 | 433.1140 | 0.12 |
| 99 | Prunin | -H2 | 14.12 | M+H | 433.1121 | 433.1129 | -2.02 |
| 100 | Semilicoisoflavone b |  | 17.29 | M+H | 353.1008 | 353.1020 | -3.17 |
| 101 | Sigmoidin-B |  | 19.47 | M-H | 355.1189 | 355.1187 | 0.55 |
| 102 | tangshenoside |  | 10.04 | M-H | 677.2307 | 677.2298 | 1.27 |
| 103 | Vanillic acid glucoside |  | 4.58 | M-H | 329.0881 | 329.0878 | 0.82 |
| 104 | Vicenin 2 |  | 8.07 | M-H | 593.1520 | 593.1512 | 1.42 |
| 105 | Vicenin I |  | 9.37 | M+FA-H | 609.1471 | 609.1461 | 1.63 |
| 106 | Vicenin III |  | 9.82 | M+H | 565.1544 | 565.1552 | -1.32 |
| 107 | Vitexin |  | 10.60 | M+H | 433.1121 | 433.1129 | -1.93 |
| 108 | Yinyanghuo D |  | 20.34 | M+H | 339.1215 | 339.1227 | -3.57 |

**Table S3. The primers sequence of RT-qPCR.**

| **Gene name** | **Targeted sequences** |
| --- | --- |
| *Pparg* | CGAAGAACCATCCGATTGAAGC (F) |
|  | CAAGGCACTTCTGAAACCGACA (R) |
| *Fat/Cd36* | TGCAGGTCAACATACTGGTCA (F) |
|  | GTCCCAGTCTCATTTAGCCACA (R) |
| *Cpt-1* | TTGCTGTCAACCTCGGACCCAA (F) |
|  | CTTCCCACCAGTCACTCACGTA (R) |
| *Cpt-2* | AACTGCTCGAGACTCTATCGTT (F) |
|  | AAGTGTCTTCAGAAACCGGACT (R) |
| *Lcad* | AGGCTTCTACTACCTCATGCAAG (F) |
|  | CCTTTCTTTGTCTCACGTAGTTCC (R) |
| *aP2* | ATGAAATCACCCCAGATGACAGG (F) |
|  | ACCAGCTTGTCACCATCTCGTC (R) |
| *β-Actin* | AGCCATGTACGTAGCCATCC (F) |
|  | CTCTCAGCTGTGGTGGTGAA (R) |

**Figure S1. Venn diagram of candidate differentially metabolites in the Control-vs.-Model and Model-vs.-JGD-H comparisons.**

**Figure S2. Inflammatory cytokines (TNF-α and IL-1β) contents of RAW264.7 cells treated with JGD (50, 100, and 200 μg/mL).** Relative to the Control group, ^##^*P* < 0.01; relative to the Model group, ^*^*P* < 0.05, ^**^*P* < 0.01.

**Figure S3. Inflammatory cytokines (TNF-α and IL-1β) contents of RAW264.7 cells treated with LG (3.125, 6.25, and 12.5 μM).** Relative to the Control group, ^##^*P* < 0.01; relative to the Model group, ^*^*P* < 0.05, ^**^*P* < 0.01, ns: *P* > 0.05.

**original data**

Figure 1B

|  | Control | Model | JGD-L | JGD-H | DXM |
| --- | --- | --- | --- | --- | --- |
| 1 | 200 | 200.83 | 203.33 | 197.5 | 199.17 |
| 8 | 248.33 | 245 | 249.17 | 244.17 | 246.67 |
| 15 | 297.5 | 283.33 | 289.17 | 291.67 | 276.67 |
| 22 | 335.83 | 300.83 | 311.67 | 312.5 | 301.67 |
| 29 | 380.83 | 317.5 | 332.5 | 330 | 321.67 |
| 36 | 401.67 | 325 | 355 | 354.17 | 337.5 |
| 43 | 436.67 | 329.17 | 372.5 | 375.83 | 348.33 |
| 50 | 452.5 | 330.83 | 388.33 | 391.67 | 364.17 |
| 57 | 471.67 | 330 | 400.83 | 403.33 | 378.33 |
| 64 | 495 | 323.33 | 419.16 | 425 | 393.33 |

Figure 1D

| Control | Model | JGD-L | JGD-H | DXM |
| --- | --- | --- | --- | --- |
| 0 | 3 | 1 | 1 | 2 |
| 0 | 3 | 2 | 1 | 2 |
| 0 | 4 | 3 | 1 | 1 |

Figure 1F

| Control | Model | JGD-L | JGD-H | DXM |
| --- | --- | --- | --- | --- |
| 0 | 3 | 3 | 1 | 2 |
| 0 | 4 | 2 | 2 | 2 |
| 0 | 4 | 3 | 1 | 1 |

Figure 1G

BALF TNF-α

| Control | Model | JGD-L | JGD-H | DXM |
| --- | --- | --- | --- | --- |
| 158.98 | 240.93 | 190.88 | 126.13 | 166.12 |
| 118.31 | 248.24 | 186.07 | 124.16 | 133.36 |
| 107.47 | 267.84 | 219.95 | 154.94 | 153.56 |
| 173 | 235 | 197.93 | 189.85 | 185.29 |
| 136.62 | 211.35 | 199.65 | 125.53 | 175.66 |
| 91.82 | 254.94 | 205.33 | 180.65 | 133.18 |

BALF IL-6

| Control | Model | JGD-L | JGD-H | DXM |
| --- | --- | --- | --- | --- |
| 44.24 | 81.93 | 81.84 | 61.41 | 79.56 |
| 38.06 | 124.33 | 101.29 | 85.56 | 60.74 |
| 45.81 | 104.92 | 106.36 | 64.01 | 61.86 |
| 47.34 | 113.88 | 92.82 | 71.67 | 87.85 |
| 82.56 | 90.76 | 97.62 | 77.72 | 85.61 |
| 70.06 | 115.63 | 90.31 | 64.46 | 82.87 |

BALF IL-1β

| Control | Model | JGD-L | JGD-H | DXM |
| --- | --- | --- | --- | --- |
| 38.29 | 76.95 | 75.05 | 63.17 | 64.93 |
| 66.63 | 88.41 | 77.11 | 58.47 | 76.95 |
| 39.2 | 102.58 | 85.93 | 51.25 | 88.18 |
| 47.63 | 82.07 | 77.18 | 52.46 | 75.54 |
| 43.81 | 84.39 | 84.85 | 61.76 | 76.13 |
| 46.26 | 96.67 | 77.6 | 59.48 | 79.17 |

Figure 2F

Pparg

| Control | Model | JGD-H |
| --- | --- | --- |
| 1 | 0.409874 | 0.820756 |
| 1 | 0.327526 | 0.6276 |
| 1 | 0.38387 | 0.713326 |
| 1 | 0.38197 | 0.534069 |
| 1 | 0.26135 | 0.8007 |
| 1 | 0.38242 | 0.740416 |

Fat/CD36

| Control | Model | JGD-H |
| --- | --- | --- |
| 1 | 0.5936 | 0.8063 |
| 1 | 0.405167 | 0.738578 |
| 1 | 0.596704 | 0.721271 |
| 1 | 0.503937 | 0.717886 |
| 1 | 0.457979 | 0.728407 |
| 1 | 0.304083 | 0.600063 |

Cpt-1

| Control | Model | JGD-H |
| --- | --- | --- |
| 1 | 0.520709 | 0.703074 |
| 1 | 0.35872 | 0.76668 |
| 1 | 0.29313 | 1.64997 |
| 1 | 0.526175 | 0.838659 |
| 1 | 0.636904 | 0.517597 |
| 1 | 0.618114 | 1.549887 |

Cpt-2

| Control | Model | JGD-H |
| --- | --- | --- |
| 1 | 0.489493 | 0.793054 |
| 1 | 0.489411 | 1.013712 |
| 1 | 0.297786 | 1.239738 |
| 1 | 0.505271 | 1.358636 |
| 1 | 0.2067 | 0.984643 |
| 1 | 0.70931 | 0.485762 |

Lcad

| Control | Model | JGD-H |
| --- | --- | --- |
| 1 | 0.566382 | 0.86444 |
| 1 | 0.771755 | 0.580185 |
| 1 | 0.582105 | 0.524785 |
| 1 | 0.454985 | 0.926912 |
| 1 | 0.333527 | 0.860749 |
| 1 | 0.6436 | 0.708811 |

Ap2

| Control | Model | JGD-H |
| --- | --- | --- |
| 1 | 0.267597 | 0.99272 |
| 1 | 0.520521 | 0.652121 |
| 1 | 0.126544 | 0.814235 |
| 1 | 0.175188 | 0.5109 |
| 1 | 0.598068 | 0.662726 |
| 1 | 0.791663 | 1.235223 |

Figure 4E

Leukotriene b4

| Control | Model | JGD-H |
| --- | --- | --- |
| 2.84391195663154e+007 | 3.2455788526802e+008 | 2.3058219e+008 |
| 1.31784958270713e+008 | 2.24268259412924e+008 | 2.11097092338761e+008 |
| 9.58296998806398e+007 | 3.03320758320153e+008 | 1.76750696394073e+008 |
| 1.58767841441697e+008 | 2.03864408312971e+008 | 1.74920689518196e+008 |
| 5.88593555028252e+007 | 1.67801473660295e+008 | 2.28822532120009e+008 |
| 2.23551243849559e+007 | 3.99192813350181e+008 | 1.46819176139928e+008 |

5s-HETE

| Control | Model | JGD-H |
| --- | --- | --- |
| 7082088.11770435 | 2.95827567098806e+007 | 1.60588013285843e+007 |
| 2.28161810314288e+007 | 8.83381550174506e+007 | 2.00003918104862e+007 |
| 1.53230625547334e+007 | 3.06111610639886e+007 | 3.45015478579209e+007 |
| 1.62826844072302e+007 | 8.60622119704183e+007 | 2.16232163914074e+007 |
| 1.33507092968396e+007 | 3.79377917137542e+007 | 3.18542799572439e+007 |
| 1.62148946512196e+007 | 3.11182305192721e+007 | 3.30417558601995e+007 |

12s-HETE

| Control | Model | JGD-H |
| --- | --- | --- |
| 2.99630826391263e+007 | 9.42717143728416e+007 | 4.29391630221299e+007 |
| 2.96967424709689e+007 | 9.83086674848369e+007 | 4.61151556108871e+007 |
| 5.85725695389533e+007 | 1.6054424781276e+008 | 1.29122011568471e+008 |
| 2.85170640625211e+007 | 9.10437558653581e+007 | 6.23794829248888e+007 |
| 3.63640352031037e+007 | 1.49279164746349e+008 | 3.96877037238413e+007 |
| 5.48767641654937e+007 | 8.90883014386333e+007 | 5.69819319097411e+007 |

Figure 5A

| PPARγ |  |  |  |
| --- | --- | --- | --- |
| β-actin |  |  |  |

Figure 5A

| Control | Model | JGD-L | JGD-H |
| --- | --- | --- | --- |
| 0.96867092 | 0.45973326 | 0.61114363 | 0.85242258 |
| 1.10417242 | 0.36423990 | 0.53616809 | 0.93595105 |
| 1.02735414 | 0.22319993 | 0.52200885 | 0.83369237 |

Figure 5B

| Control | 1.5625 | 3.125 | 6.25 | 12.5 | 25 | 50 | 100 | 200 | 400 |
| --- | --- | --- | --- | --- | --- | --- | --- | --- | --- |
| 100 | 108.39 | 97.16 | 102.9 | 105.29 | 95.35 | 109.87 | 112.71 | 104.77 | 86.77 |
| 100 | 102.52 | 98.9 | 106.84 | 103.74 | 107.81 | 91.1 | 119.1 | 102.65 | 83.1 |
| 100 | 105.61 | 105.42 | 100.32 | 110.39 | 105.87 | 114.84 | 107.55 | 106.13 | 83.1 |

Figure 5C

| Control | LPS | 50 | 100 | 200 |
| --- | --- | --- | --- | --- |
| 1 | 0.323754309 | 0.42192444 | 0.815703968 | 1.168730909 |
| 1 | 0.356009572 | 0.562160751 | 0.650189643 | 1.056852945 |
| 1 | 0.496946799 | 0.362270659 | 0.744184055 | 1.165517988 |

Figure 5D

| PPARγ |  |  |  |
| --- | --- | --- | --- |
| β-actin |  |  |  |

Figure 5D

| Control | LPS | 50 | 100 | 200 |
| --- | --- | --- | --- | --- |
| 1 | 0.471614882 | 0.552125643 | 0.684403006 | 0.78646951 |
| 1 | 0.429608979 | 0.597857273 | 0.757208316 | 1.069063608 |
| 1 | 0.352425885 | 0.481549219 | 0.741439962 | 0.921631411 |

Figure 8B

| Control | 1.5625 | 3.125 | 6.25 | 12.5 | 25 | 50 | 100 | 200 | 400 |
| --- | --- | --- | --- | --- | --- | --- | --- | --- | --- |
| 100 | 106.62 | 110.44 | 101.67 | 102.19 | 94.73 | 90.47 | 88.91 | 91.69 | 75.97 |
| 100 | 110.09 | 104.88 | 99.59 | 99.07 | 98.55 | 90.99 | 88.3 | 88.73 | 72.85 |
| 100 | 105.32 | 103.32 | 103.15 | 100.98 | 95.59 | 92.64 | 93.16 | 89.95 | 75.19 |

Figure 8C

TNF-α

| Control | LPS | LPS+LG | LPS+GW9662 | LPS+LG+GW9662 |
| --- | --- | --- | --- | --- |
| 418.75 | 36637.5 | 10606.25 | 44950 | 22575 |
| 450 | 37793.75 | 10106.25 | 46450 | 22450 |
| 481.25 | 36325 | 9450 | 43106.25 | 25700 |

IL-1β

| Control | LPS | LPS+LG | LPS+GW9662 | LPS+LG+GW9662 |
| --- | --- | --- | --- | --- |
| 1841.142857 | 2092.571429 | 1898.285714 | 2269.714286 | 1966.857143 |
| 1852.571429 | 2115.428571 | 1878.285714 | 2252.571429 | 2001.142857 |
| 1841.142857 | 2001.142857 | 1824 | 2226.857143 | 2012.571429 |

Figure 8D

| Control | LPS | LPS+LG | LPS+GW9662 | LPS+LG+GW9662 |
| --- | --- | --- | --- | --- |
| 1.047461889 | 0.557186907 | 1.105435452 | 0.384657278 | 0.731890644 |
| 0.987384979 | 0.635055904 | 0.909492489 | 0.406780124 | 0.617801224 |
| 0.964103634 | 0.53014164 | 0.974267212 | 0.306566833 | 0.627464307 |

Figure 8E

| PPARγ |  |  |  |
| --- | --- | --- | --- |
| β-actin |  |  |  |

Figure 8E

| Control | LPS | LPS+LG | LPS+GW9662 | LPS+LG+GW9662 |
| --- | --- | --- | --- | --- |
| 1.108256978 | 0.579366586 | 0.846286107 | 0.305393725 | 0.621774301 |
| 0.936191213 | 0.605769961 | 0.912856814 | 0.307164432 | 0.589952328 |
| 0.97316818 | 0.630905437 | 0.947088288 | 0.329929412 | 0.623865163 |

Figure 8F

COX-2

| Control | LPS | LPS+LG | LPS+GW9662 | LPS+LG+GW9662 |
| --- | --- | --- | --- | --- |
| 1010 | 1536.470588 | 1410 | 2083.529412 | 1962.941176 |
| 780.5882353 | 1533.529412 | 1274.705882 | 2110 | 1933.529412 |
| 995.2941176 | 1742.352941 | 1257.058824 | 2127.647059 | 1657.058824 |
| 839.4117647 | 1448.235294 | 1121.764706 | 2360 | 1654.117647 |
| 1115.882353 | 1662.941176 | 1162.941176 | 2127.647059 | 1221.764706 |
| 1060 | 930.588235 | 1027.647059 | 2042.352941 | 1357.058824 |

5-LOX

| Control | LPS | LPS+LG | LPS+GW9662 | LPS+LG+GW9662 |
| --- | --- | --- | --- | --- |
| 2.941 | 6.47 | 3.529 | 8.201 | 5.294 |
| 3.529 | 5.294 | 3.918 | 7.058 | 5.882 |
| 2.882 | 5.294 | 3.927 | 7.058 | 4.188 |
| 3.117 | 5.294 | 2.168 | 7.647 | 6.47 |
| 2.706 | 2.941 | 3.927 | 9.411 | 5.294 |
| 2.882 | 5.882 | 3.842 | 7.647 | 7.658 |

Figure 9B

TNF-α

| Control | Model | JGD-L | JGD-H | DXM |
| --- | --- | --- | --- | --- |
| 115.6209662 | 239.222674 | 180.7636208 | 158.8606657 | 192.8218085 |
| 117.6947383 | 247.3834654 | 180.5985237 | 156.3950773 | 203.0361527 |
| 121.7122208 | 233.899363 | 180.5434978 | 154.294909 | 192.5191982 |
| 121.9203153 | 240.1350969 | 181.4242976 | 150.4670978 | 198.1608829 |
| 113.2052159 | 244.1421227 | 180.2684164 | 159.6326785 | 197.735274 |
| 114.7922661 | 234.5287875 | 183.5194897 | 144.5317581 | 195.9737123 |

IL-1β

| Control | Model | JGD-L | JGD-H | DXM |
| --- | --- | --- | --- | --- |
| 48.99735826 | 111.991275 | 88.33642244 | 71.39592452 | 87.22103204 |
| 43.69109492 | 112.3086877 | 66.91929427 | 65.8993466 | 83.03784931 |
| 44.98542 | 112.1499224 | 64.4739266 | 67.16052029 | 94.96906879 |
| 52.19892716 | 110.2539377 | 72.46098271 | 69.66028594 | 88.6164494 |
| 46.49800306 | 108.8430942 | 62.9488862 | 72.0213974 | 78.89851231 |
| 53.78876132 | 114.2230703 | 64.35590818 | 76.28811967 | 88.26648941 |

IL-6

| Control | Model | JGD-L | JGD-H | DXM |
| --- | --- | --- | --- | --- |
| 45.41176471 | 112.505882353 | 78.83529411 | 50.11764706 | 65.482352941 |
| 47.76470588 | 102.152941176 | 73.364705882 | 43.05882353 | 64.541176471 |
| 57.17647059 | 123.329411765 | 76 | 66.58823529 | 65.247058824 |
| 45.41176471 | 127.741176471 | 85.1176471 | 54.82352941 | 72.270588235 |
| 59.52941176 | 92.623529412 | 84.82352941 | 52.47058824 | 62.388235294 |
| 57.17647059 | 102.741176471 | 80.70588235 | 54.82352941 | 72.858823529 |
